# Supplementary material for: Wipi3 is essential for alternative autophagy and its loss causes neurodegeneration
Source: Nat Commun. 2020 Oct 20;11:5311. doi: 10.1038/s41467-020-18892-w (PMC7576787; doi:10.1038/s41467-020-18892-w)
Supplement: Supplementary file 1 — Supplementary Information [file 41467_2020_18892_MOESM1_ESM.pdf]

Suppl. Fig. 1 Yamaguchi et al.

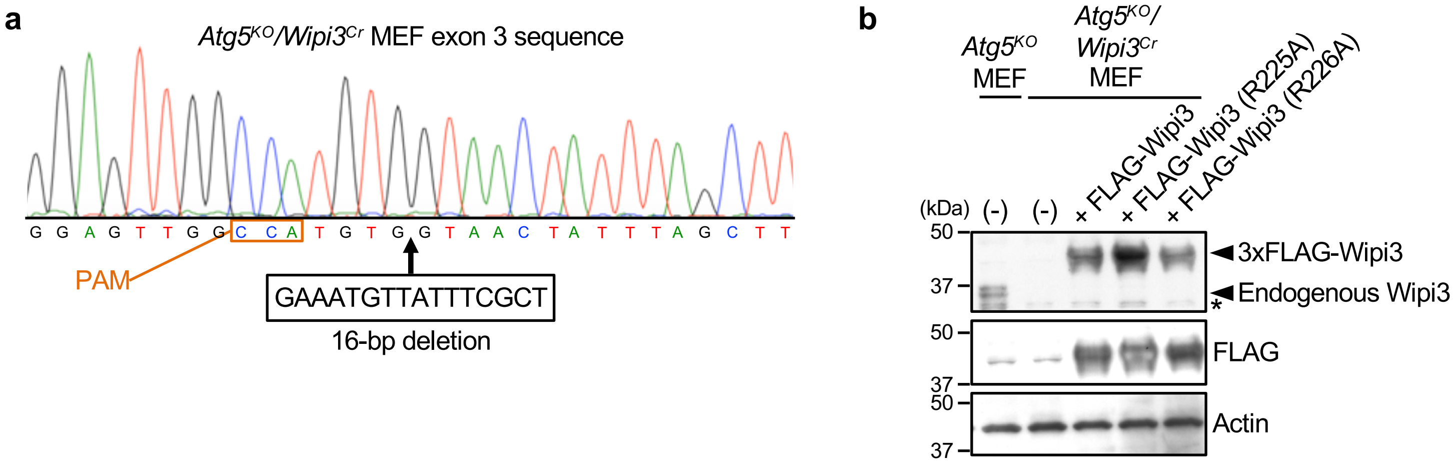

Supplementary Figure 1. Generation of *Atg5^{KO}/Wipi3^{Cr}* MEFs

Atg5^{KO}/Wipi3^{Cr} MEFs were generated from *Atg5^{KO}* MEFs using the CRISPR/Cas9 system. A 16-bp deletion in *wipi3* (third exon) was confirmed by genomic sequencing. The deleted nucleotide sequence and proto-spacer adjacent motif (PAM) are indicated below the sequence. In (**b**), the deletion of Wipi3 and the expression of Wipi3 mutants in *Atg5^{KO}/Wipi3^{Cr}* MEFs were confirmed by western blot analysis. The asterisk indicates a non-specific band. Source data are provided as a Source Data file.

Suppl. Fig. 2 Yamaguchi et al.

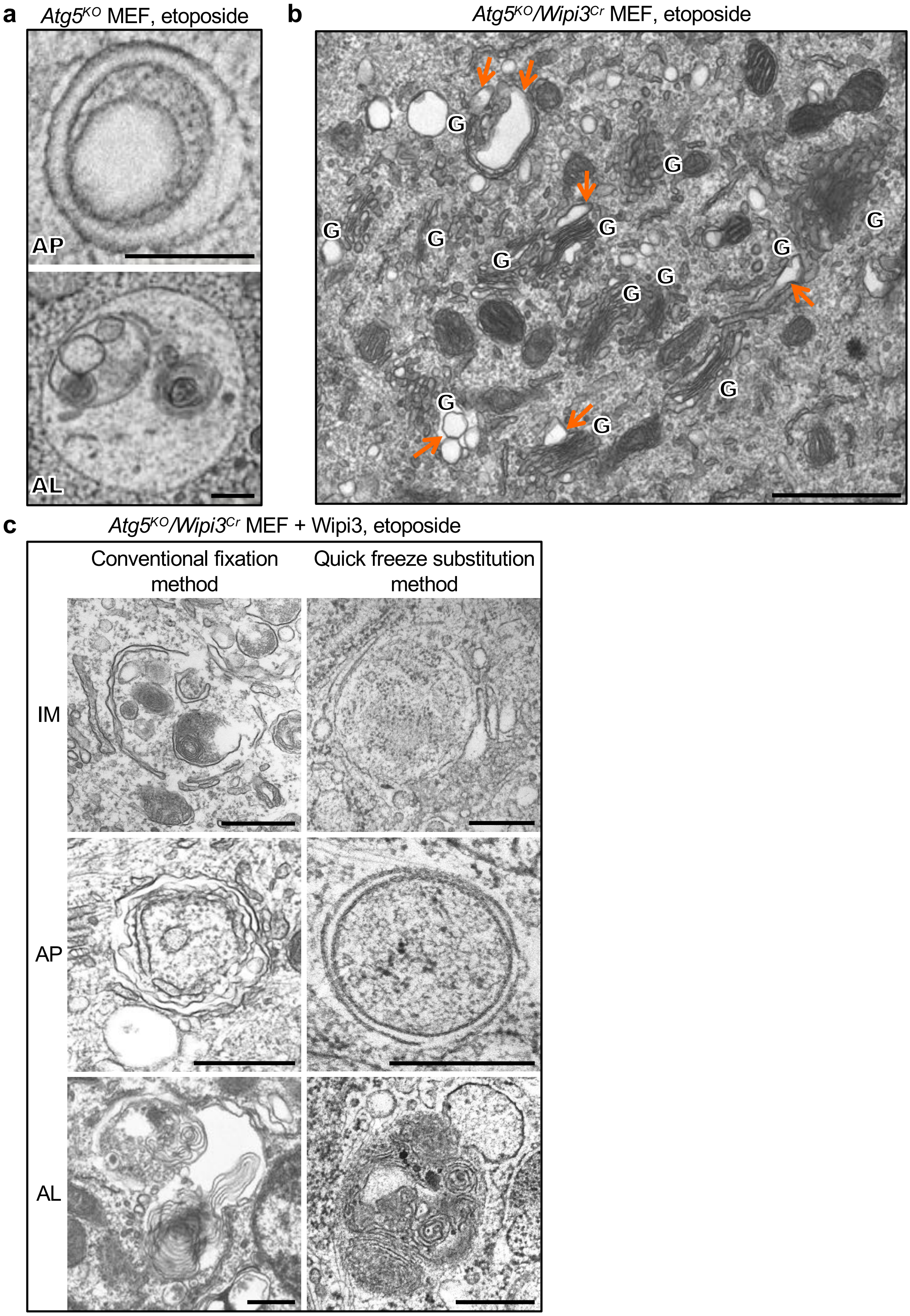

Supplementary Figure 2. Effects of Wipi3 on Golgi morphology and alternative autophagy

The indicated MEFs were treated with etoposide (10 µM for 12 hr), and analyzed by EM. (**a**) Representative autophagosome (AP) and autolysosome (AL) were observed in *Atg5^{KO}* MEFs (quick freeze substitution method). Bars = 0.2 µm. (**b**) A magnified image of the dashed square in Fig. 2b is shown. Bar = 1 µm. Abnormally swollen Golgi structures (arrows) were observed in *Atg5^{KO}/Wipi3^{Cr}* MEFs. "G" indicates the Golgi apparatus. (**c**) Representative isolation membranes (IM), autophagosome (AP), and autolysosome (AL) were observed in Wipi3-expressed *Atg5^{KO}/Wipi3^{Cr}* MEFs. Bars = 0.5 µm.

Suppl. Fig. 3 Yamaguchi et al.

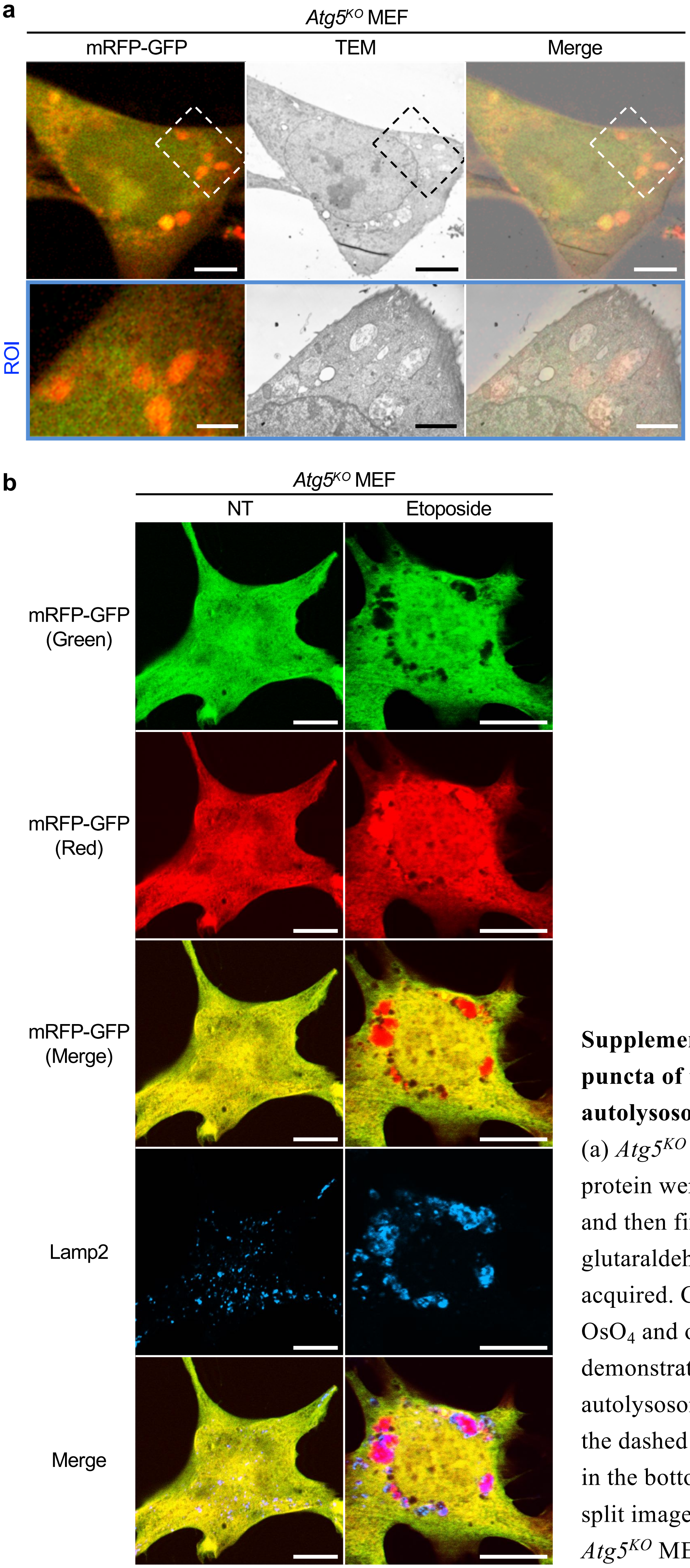

Supplementary Figure 3. Confirmation of red puncta of the mRFP-GFP tandem protein as autolysosomes by CLEM analysis

(a) *Atg5*^{KO} MEFs expressing the mRFP-GFP tandem protein were treated with etoposide (10 μM) for 18 hr, and then fixed with 0.75% paraformaldehyde/1.5% glutaraldehyde, and mRFP-GFP images were acquired. Cells were subsequently fixed with 1% OsO_4 and observed by EM. CLEM analysis demonstrated that red puncta were merged with autolysosomes. Bars = 5 μm. ROIs are indicated by the dashed squares and magnified images are shown in the bottom panels. Bars = 2 μm. (**b**) Magnified and split images of untreated and etoposide-treated *Atg5*^{KO} MEFs in Fig. 2d are shown.

Suppl. Fig. 4 Yamaguchi et al.

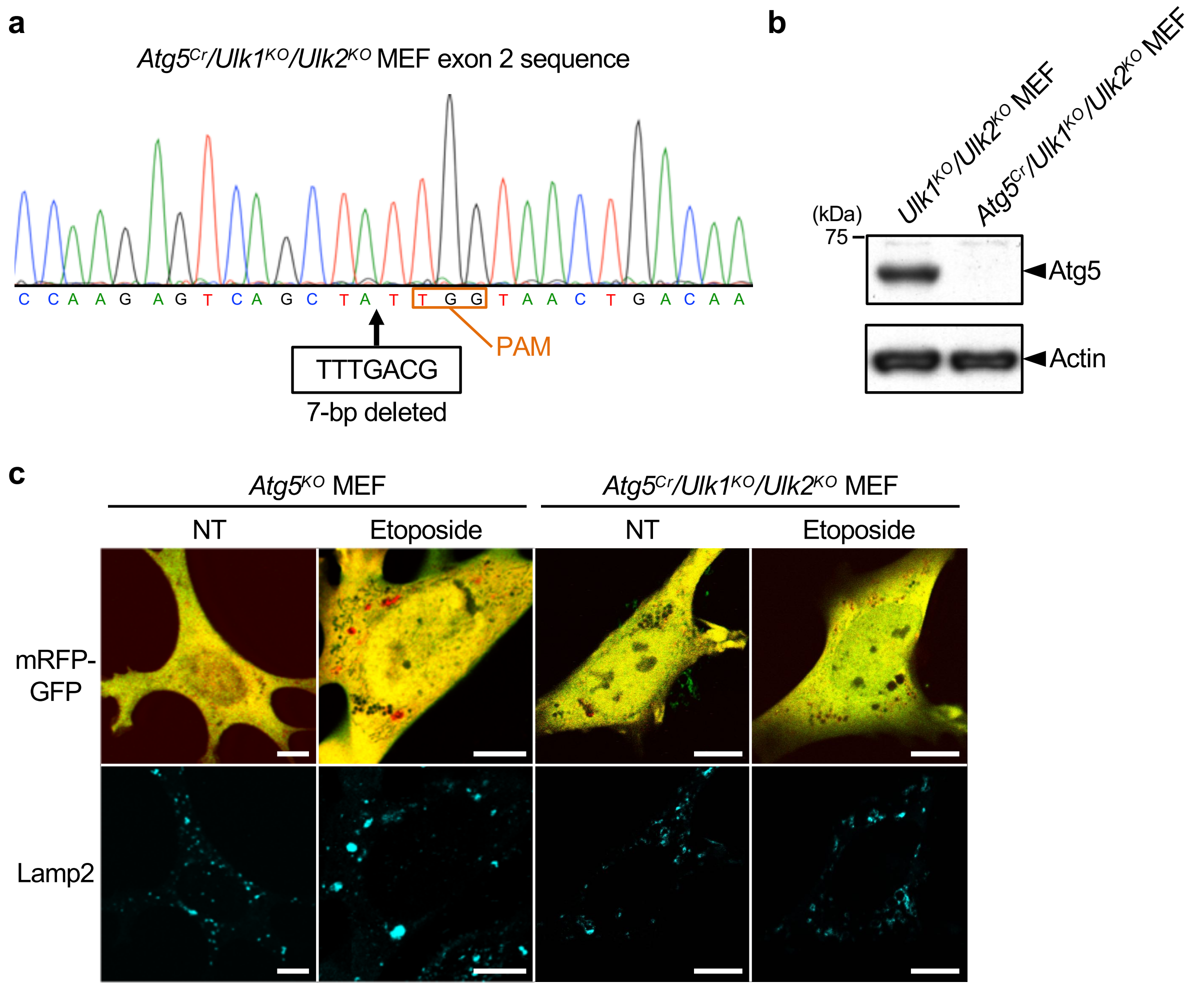

Supplementary Figure 4. No induction of alternative autophagy in Atg5/Ulk1/Ulk2 triple-knockout MEFs

(**a**, **b**) *Atg5^{Cr}/Ulk1^{KO}/Ulk2^{KO}* MEFs were generated from *Ulk1^{KO}/Ulk2^{KO}* MEFs using the CRISPR/Cas9 system. A 7-bp deletion in *atg5* (second exon) was confirmed by genomic sequencing. The deleted nucleotide sequence and PAM are indicated below the sequence. In (**b**), the deletion of Atg5 was confirmed by western blot analysis. (**c**) The indicated MEFs expressing mRFP-GFP were left untreated (NT) or were treated with etoposide (10 µM; 18 hr) and observed using confocal microscopy. Bars = 10 µm. Red puncta (autolysosomes) were observed in *Atg5^{KO}* MEFs, but not *Atg5^{Cr}/Ulk1^{KO}/Ulk2^{KO}* MEFs, upon etoposide treatment. Source data are provided as a Source Data file.

Suppl. Fig. 5 Yamaguchi et al.

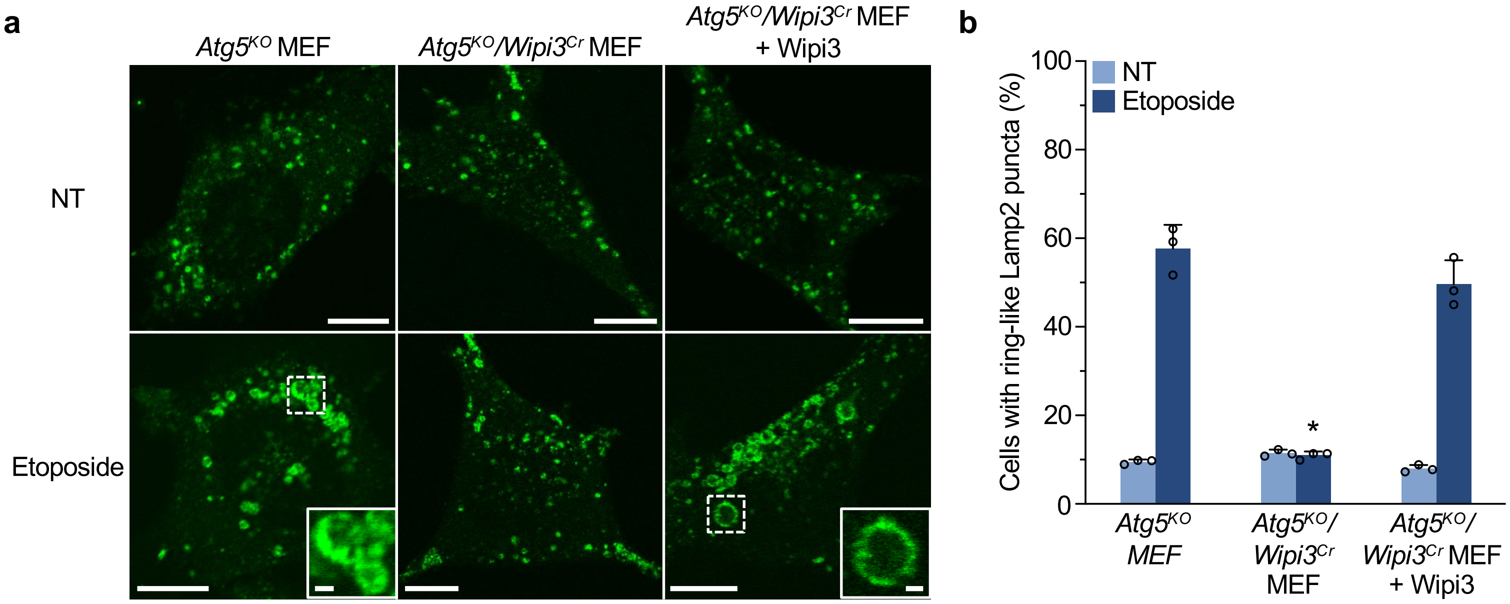

Supplementary Figure 5. Wipi3 is essential for etoposide-induced alternative autophagy

The indicated MEFs were treated with etoposide (10 µM for 18 hr), and then stained with an anti-Lamp2 antibody. Representative images are shown in (**a**). Bars = 10 µm. Magnified images of the dashed squares are shown in the inset. Bars = 1 µm. Ring-like Lamp2 puncta demonstrate autolysosomes. (**b**) The populations of MEFs with ring-like Lamp2 puncta were calculated. Data are shown as the mean ± SD (n > 110 cells examined over 3 independent experiments). Comparisons were performed using one-way ANOVA followed by the Tukey post-hoc test. *p < 0.01 vs the value of etoposide-treated *Atg5^{KO}* MEFs (exact p value cannot be described since the value is too small [p<0.0001]). Source data are provided as a Source Data file.

Suppl. Fig. 6 Yamaguchi et al.

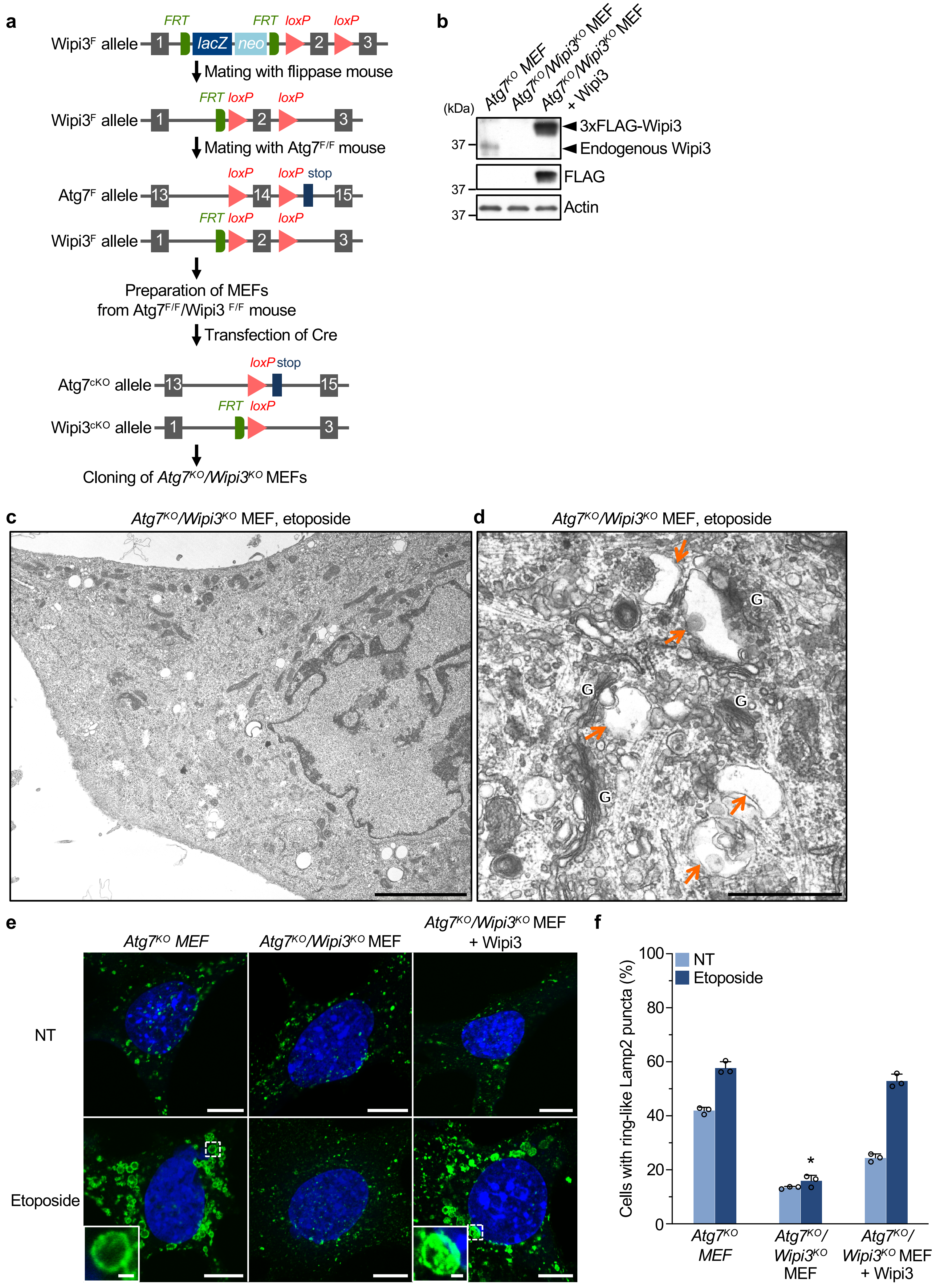

Supplementary Figure 6. Lack of etoposide-induced alternative autophagy in *Atg7^{KO}/Wipi3^{KO}* MEFs

(**a**, **b**) Generation of *Atg7^{KO}/Wipi3^{KO}* MEFs. (**a**) Schema of MEF generation. *Atg7^{KO}/Wipi3^{KO}* MEFs were generated by the transfection of Cre recombinase in *Atg7$^{F/F}$/Wipi3$^{F/F}$* MEFs. (**b**) The deletion of Wipi3 in the indicated cells was confirmed by western blot analysis using the indicated antibodies. (**c**, **d**) *Atg7^{KO}/Wipi3^{KO}* MEFs were treated with etoposide (10 μM for 18 hr), and analyzed using EM. (**c**) No autophagic structures were observed. (**d**) The Golgi apparatus formed ministacks and some Golgi membranes were swollen (arrows). "G" indicates the Golgi apparatus. Bar = 5 μm (**c**) and 1 μm (**d**). (**e**, **f**) The indicated MEFs were left untreated (NT) or treated with etoposide (10 μM for 18 hr), and were stained with an anti-Lamp2 antibody. Representative images are shown. Bars = 10 μm. Magnified images of the dashed squares are shown in the inset. Bars = 1 μm. The ring-like Lamp2 puncta demonstrate autolysosomes. (**f**) The populations of MEFs with ring-like Lamp2 puncta were calculated. Data are shown as the mean ± SD (n > 204 cells examined over 3 independent experiments). Comparisons were performed using one-way ANOVA followed by the Tukey post-hoc test. *$p < 0.01$ vs the value of etoposide-treated *Atg7^{KO}* MEFs (exact *p* value cannot be described since the value is too small [$p<0.0001$]). Source data are provided as a Source Data file.

Suppl. Fig. 7 Yamaguchi et al.

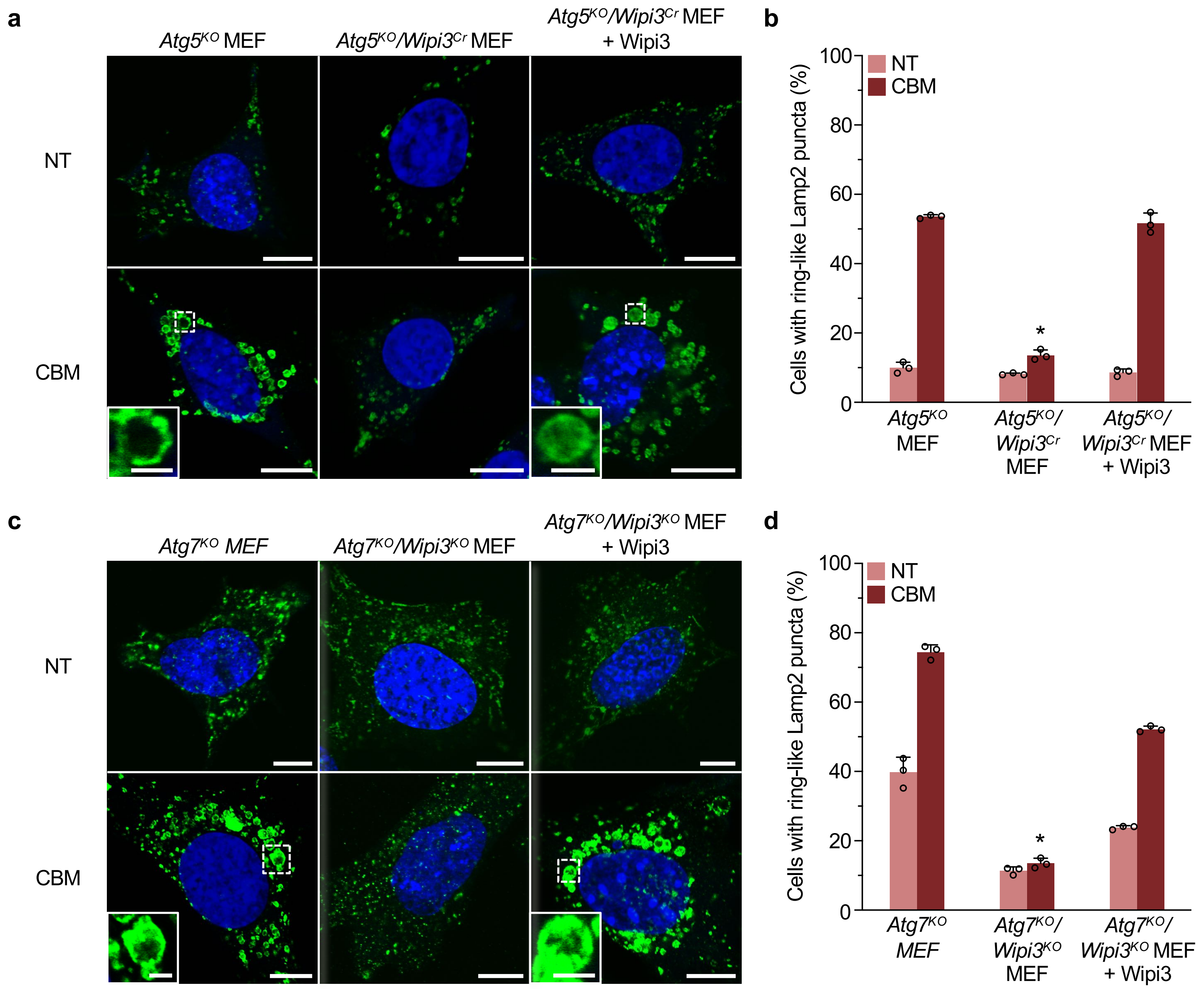

Supplementary Figure 7. Involvement of Wipi3 in CBM-induced alternative autophagy

(**a**, **c**) The indicated MEFs were left untreated (NT) or treated with 1,3-cyclohexanebis (methylamine) (CBM, 2 mM for 24 hr), and were stained with an anti-Lamp2 antibody. Representative images are shown; bars = 10 μm. Magnified images of the dashed squares are shown in the inset; bars = 2 μm. Ring-like Lamp2 puncta are autolysosomes. (**b**, **d**) The population of MEFs with ring-like Lamp2 puncta were calculated. Data are shown as the mean ± SD (**b**: n > 202 cells; **d**: n > 201 cells examined over 3 independent experiments). Comparisons were performed using one-way ANOVA followed by the Tukey post-hoc test. In (**b**) and (**d**), *p < 0.01 vs the value of etoposide-treated *Atg5^{KO}* MEFs and vs the value of etoposide-treated *Atg7^{KO}* MEFs, respectively (exact p value cannot be described since the value is too small [p<0.0001]). Source data are provided as a Source Data file.

Suppl. Fig. 8 Yamaguchi et al.

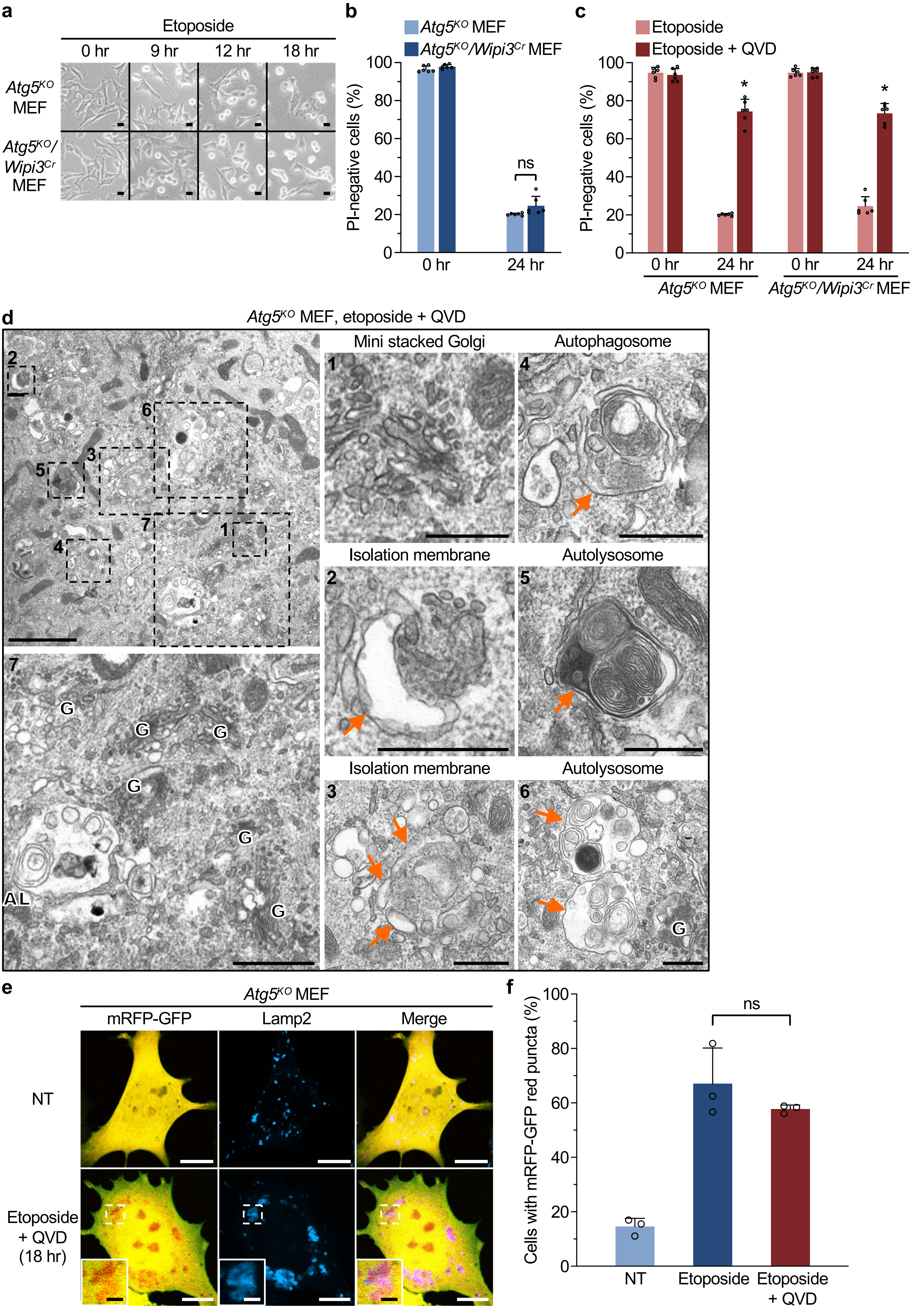

Supplementary Figure 8. No involvement of apoptosis in Wipi3-mediated alternative autophagy
(**a**) The indicated MEFs were treated with 10 µM etoposide, and observed using a phase-contrast microscope. Bars = 20 µm. (**b**) The extent of cell death was analyzed by propidium iodide (PI) staining and flow cytometry. PI-positive cells indicate plasma membrane-ruptured dead cells. Data are shown as the mean ± SD (n = 6). (**c**) Inhibition of etoposide-induced apoptosis by a caspase inhibitor. The indicated MEFs were treated with etoposide (10 µM) with or without q-VD-OPh (QVD; 100 µM), and the extent of cell death was analyzed using PI. Data are shown as the mean ± SD (n = 6). (**d**) No effects of the caspase inhibitor on the morphology of etoposide-treated $Atg5^{KO}$ MEFs. $Atg5^{KO}$ MEFs were treated with etoposide (10 µM) and q-VD-OPh (100 µM) for 12 hr, and analyzed using EM. ROIs are indicated by dashed squares and magnified images are shown in the right panels. Arrows indicate autophagic structures. "G" and "AL" indicate the Golgi apparatus and autolysosome, respectively. Scale bar = 2 µm (left upper panel), 0.5 µm (panels 1–6), and 1 µm (panel 7). (**e**, **f**) $Atg5^{KO}$ MEFs expressing a mRFP-GFP tandem protein were left untreated or treated with etoposide (10 µM) with or without q-VD-OPh (100 µM) for 18 hr. Then, cells were immunostained with an anti-Lamp2 antibody (**e**). Red puncta indicate acidic compartments and were merged with Lamp2 fluorescence. Bars = 10 µm. Magnified images are shown in the inset. Bars = 2 µm. In (**f**), the populations of cells with red puncta per attached cells were calculated. Data are shown as the mean ± SD (n > 29 cells examined over 3 independent experiments). In (**b**, **c**, **f**), comparisons were performed using one-way ANOVA followed by the Tukey post-hoc test. In (**b**, **f**), "ns" indicates no significant difference (p = 0.4550 [**b**] and 0.3797 [**f**]). In (**c**), *p < 0.01 vs the value of etoposide-treated indicated MEFs without qVD-fmk (exact p values cannot be described since the value is too small [p<0.0001]). Source data are provided as a Source Data file.

Suppl. Fig. 9 Yamaguchi et al.

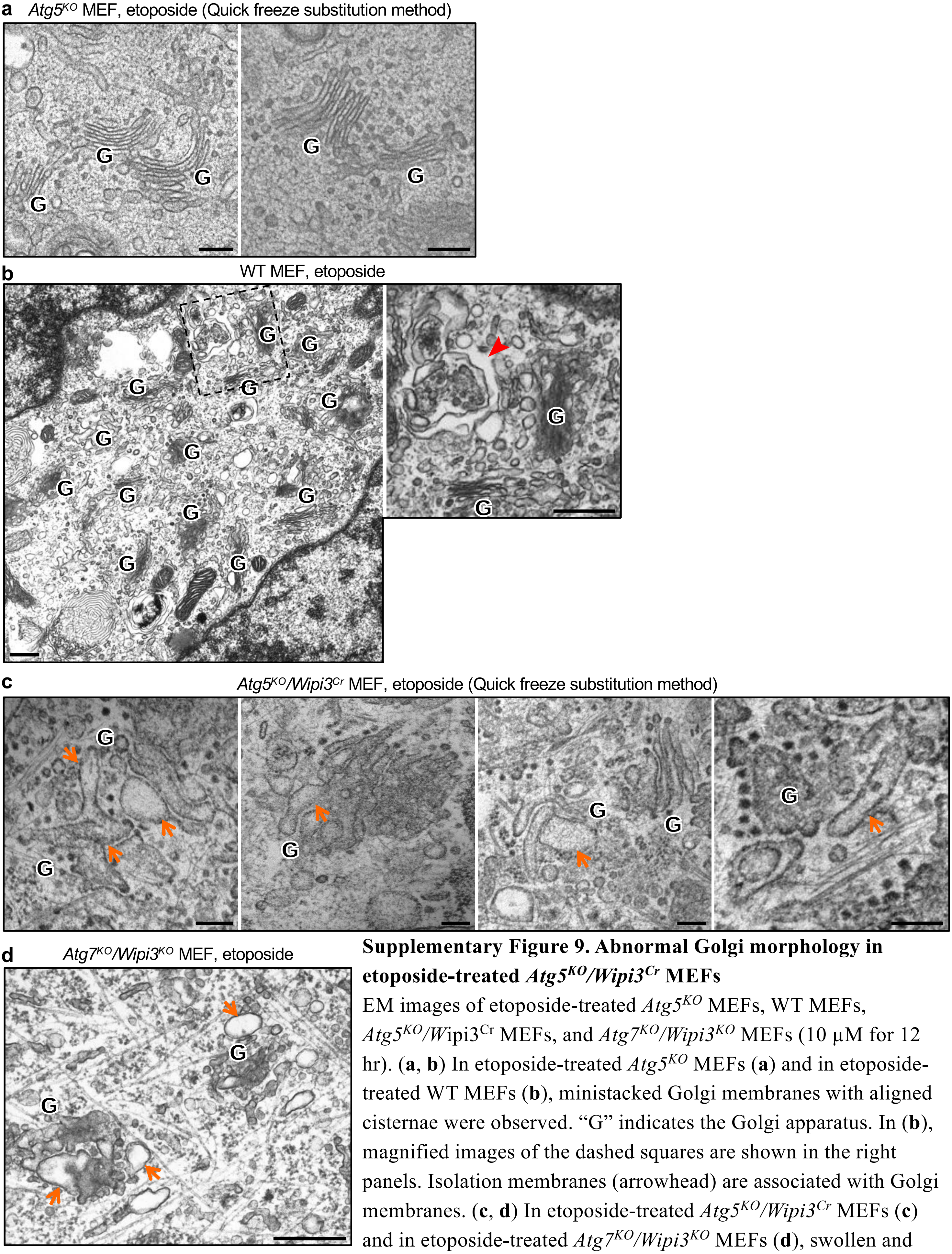

Supplementary Figure 9. Abnormal Golgi morphology in etoposide-treated *Atg5*^{KO}/*Wipi3*^{Cr} MEFs

EM images of etoposide-treated *Atg5*^{KO} MEFs, WT MEFs, *Atg5*^{KO}/*W*ipi3^{Cr} MEFs, and *Atg7*^{KO}/*Wipi3*^{KO} MEFs (10 μM for 12 hr). (**a**, **b**) In etoposide-treated *Atg5*^{KO} MEFs (**a**) and in etoposide-treated WT MEFs (**b**), ministacked Golgi membranes with aligned cisternae were observed. "G" indicates the Golgi apparatus. In (**b**), magnified images of the dashed squares are shown in the right panels. Isolation membranes (arrowhead) are associated with Golgi membranes. (**c**, **d**) In etoposide-treated *Atg5*^{KO}/*Wipi3*^{Cr} MEFs (**c**) and in etoposide-treated *Atg7*^{KO}/*Wipi3*^{KO} MEFs (**d**), swollen and rod-shaped Golgi membranes (arrows) were observed and some of them were disintegrated into vesicles. "G" indicates the Golgi apparatus. Bars = 0.2 μm (**a**, **c**) and 0.5 μm (**b**, **d**).

Suppl. Fig. 10 Yamaguchi et al.

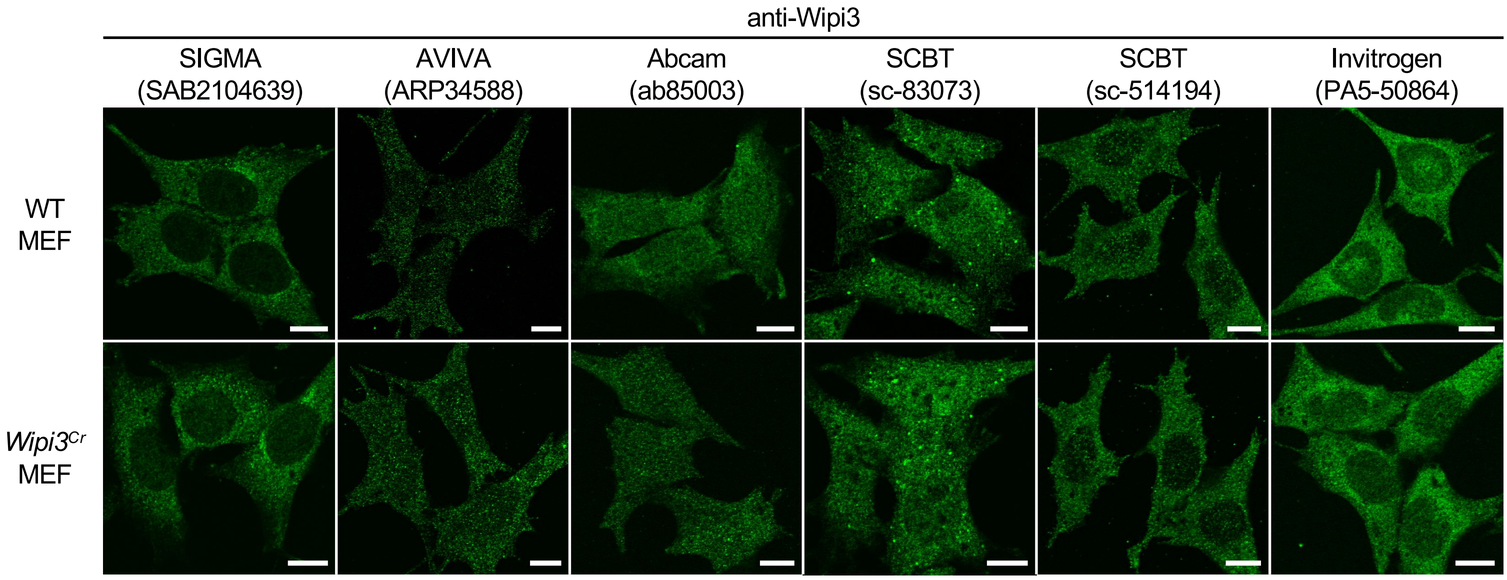

Supplementary Figure 10. No useful antibodies for Wipi3 immunofluoresence

The indicated MEFs were imunostained with the indicated anti-Wipi3 antibodies. However, we did not find any difference between WT MEF and *Wipi3*^{Cr} MEFs. Bars = 10 μm.

Suppl. Fig. 11 Yamaguchi et al.

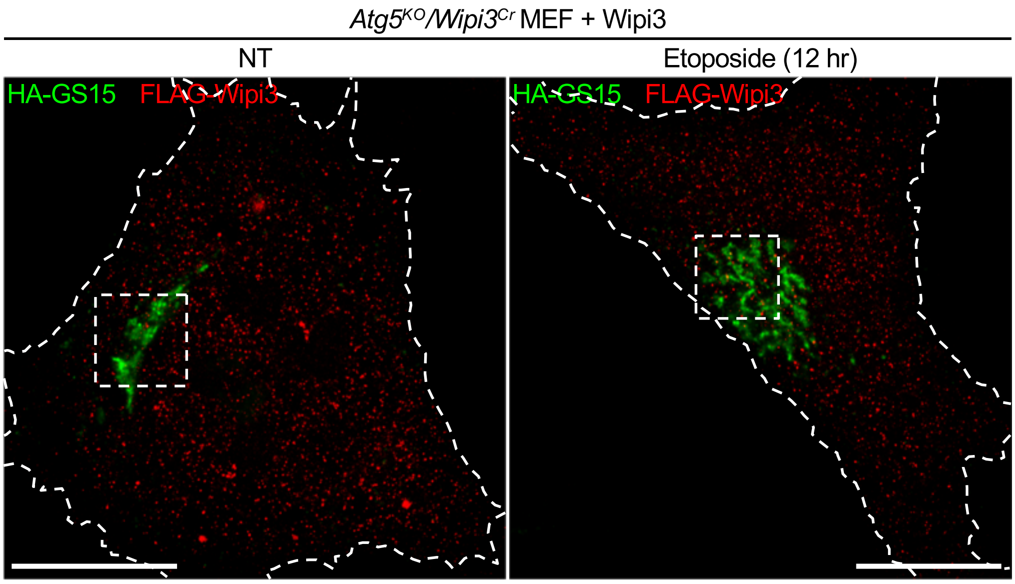

Supplementary Figure 11. Association of Wipi3 with the *trans*-Golgi upon etoposide treatment
Atg5^KO/Wipi3^Cr MEFs expressing Flag-Wipi3 and HA-GS15 were left untreated (NT) or were treated with etoposide (10 μM) at 12 hr. Then, cells were immunostained and observed by STED. Red and green signals indicate Flag-Wipi3 and HA-GS15 (*trans*-Golgi), respectively. ROIs are indicated by dashed squares and magnified images are shown in Fig. 3c. Dashed lines indicate the cell shape. Bars = 10 μm.

Suppl. Fig. 12 Yamaguchi et al.

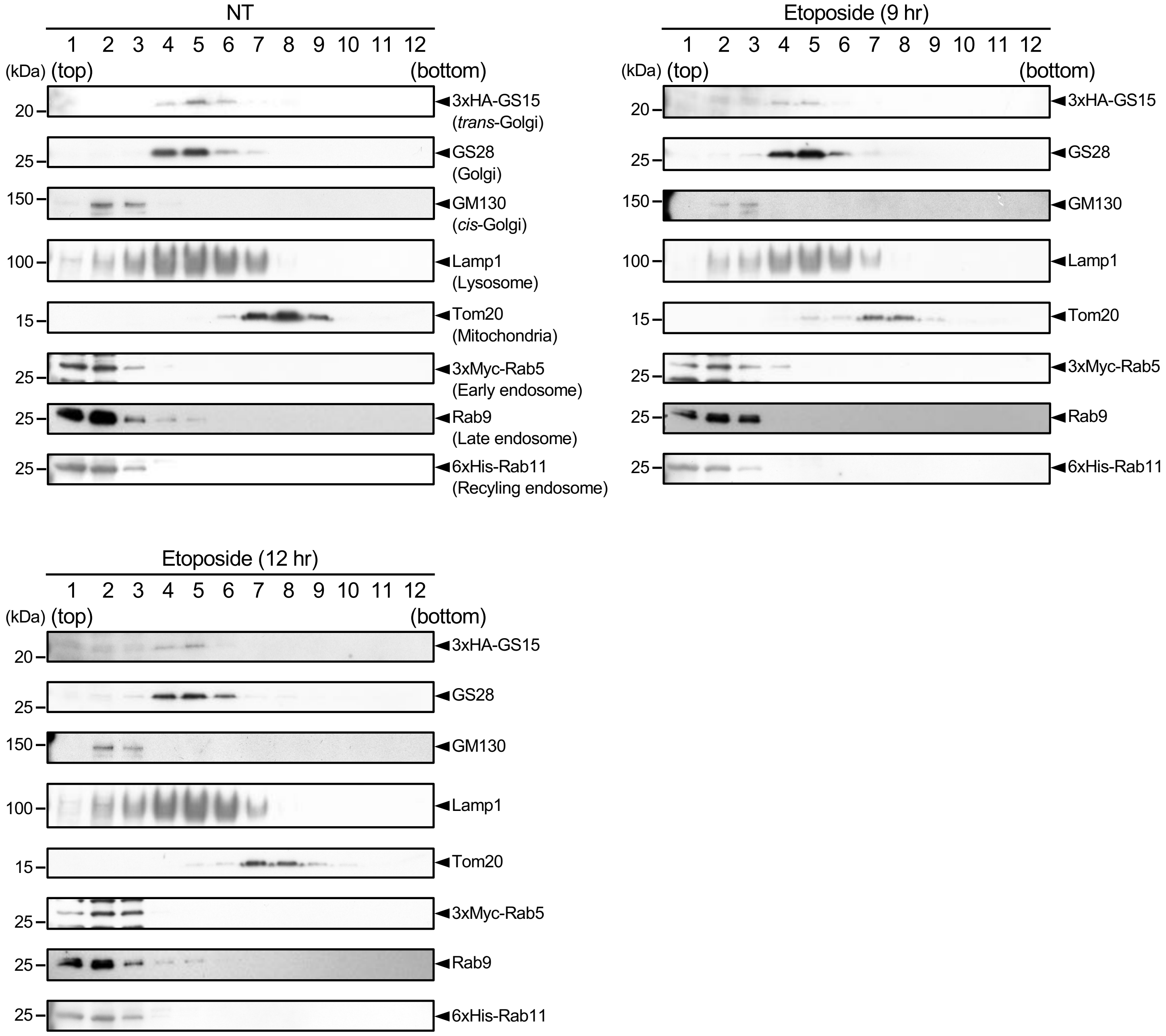

Supplementary Figure 12. Successful fractionation of *trans*-Golgi membranes

Atg5^{KO} MEFs were left untreated (NT) or treated with etoposide (10 μM), lysed in isotonic buffer at the indicated times, and fractionated by sucrose-gradient centrifugation. The fraction numbers are displayed from lightest to heaviest. The expression of each molecule was analyzed by western blotting. Fractions 1–3: endosome/*cis*-Golgi; fractions 4–6: *trans*-Golgi; and fractions 7–9: mitochondria. Source data are provided as a Source Data file.

Suppl. Fig. 13 Yamaguchi et al.

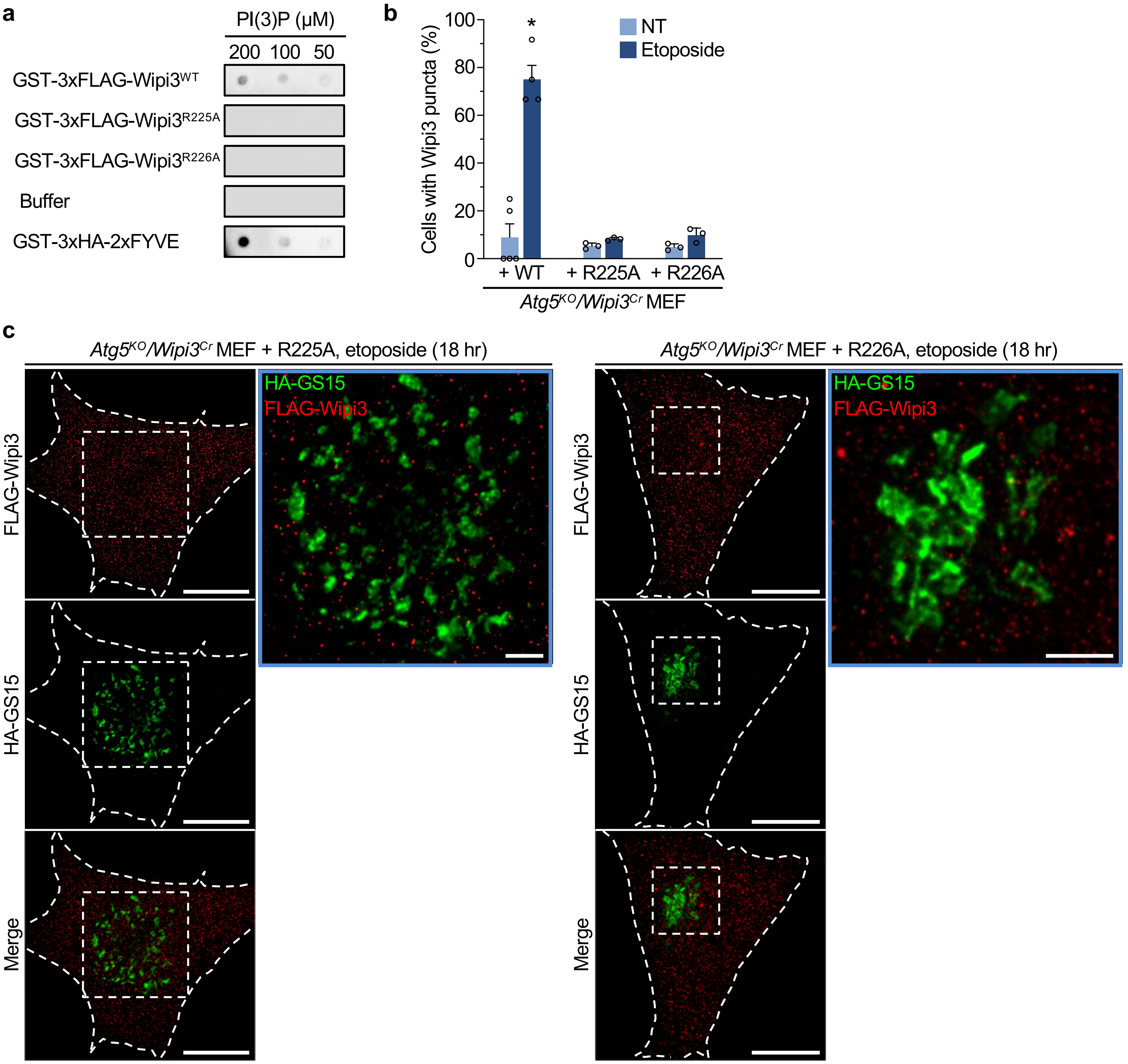

Supplementary Figure 13. Mutant Wipi3s were not associated with PI3P and *trans*-Golgi
(**a**) The binding of GST-Wipi3 and its mutants to PI(3)P was analyzed by dot-blotting using an antibody to the GST. GST-HA-FYVE was used as a positive control. (**b**) The populations of MEFs with Wipi3 puncta (larger than 1 μm) were obtained from the images in Figure 4f. Data are shown as the mean ± SEM (n > 35 cells examined over 3 independent experiments). (**c**) Similar experiments to Fig. 3c were performed by the transfection of two Flag-Wipi3 mutants at 18 hr. ROIs are indicated by the dashed squares and magnified images are shown in the right panels. Dashed lines indicate the cell shape. Bars =10 μm (left panels) and 2 μm (right panels). In (**b**), comparisons were performed using one-way ANOVA followed by the Tukey post-hoc test. *$p < 0.01$ vs the value of untreated wild-type Wipi3-expressing MEFs (exact p value cannot be described since the value is too small [$p<0.0001$]). Source data are provided as a Source Data file.

Suppl. Fig. 14 Yamaguchi et al.

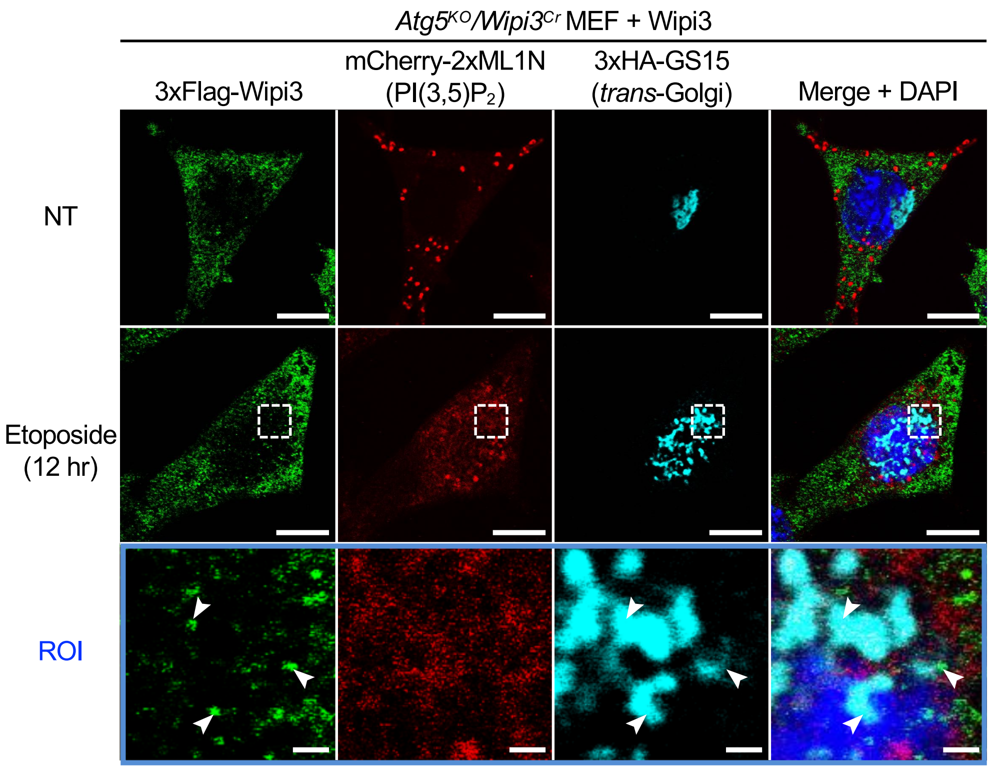

Supplementary Figure 14. No association of Wipi3 with PI(3, 5)P_2 in etoposide-treated MEFs
$Atg5^{KO}$/$Wipi3^{Cr}$ MEFs expressing Flag-Wipi3, HA-GS15, and mCherry-ML1N were left untreated (NT) or were treated with etoposide (10 μM) for 12 hr. Green, red, and blue signals indicate Flag-Wipi3, PI(3, 5)P_2, and HA-GS15, respectively. Bars =10 μm. ROIs are indicated by dashed squares and magnified images are shown in the bottom panels. Bars = 1 μm. Arrowheads indicate colocalization of Wipi3 and GS15, which were not merged with PI(3, 5)P_2.

Suppl. Fig. 15 Yamaguchi et al.

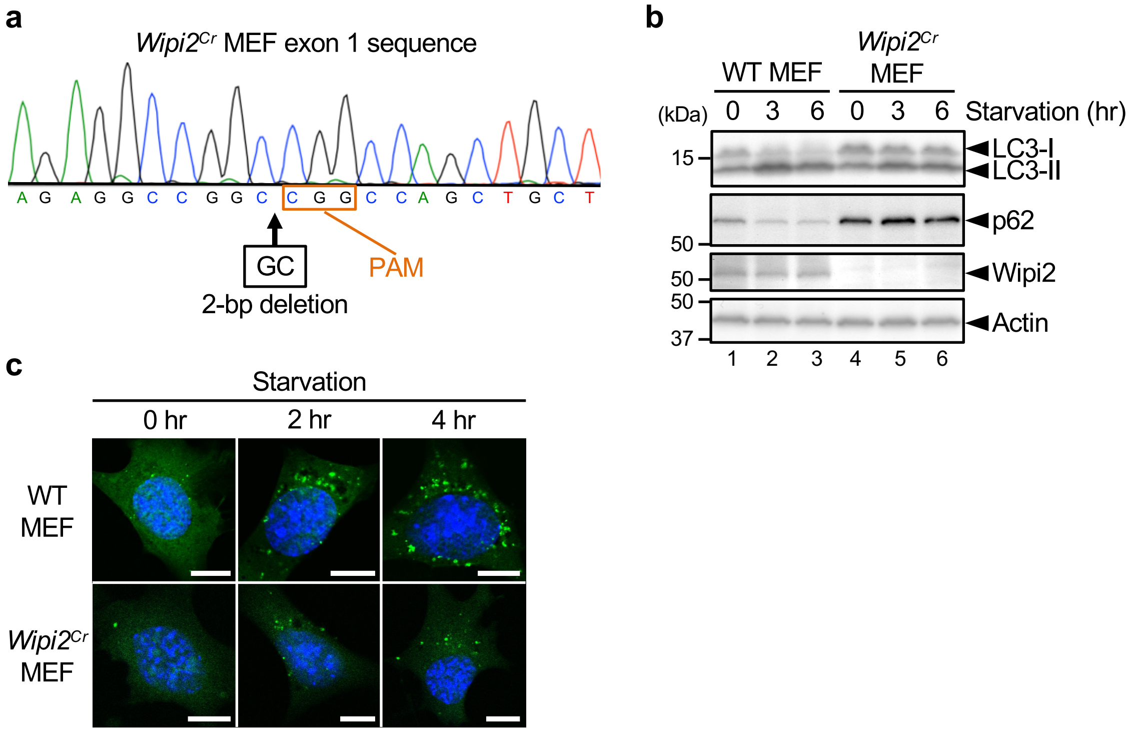

Supplementary Figure 15. Involvement of Wipi2 in canonical autophagy

(**a**) *Wipi2*^{Cr} MEFs were generated from WT MEFs using the CRISPR/Cas9 system. A 2-bp deletion in *wipi2* (first exon) was confirmed by genomic sequencing. The deleted nucleotide sequence and PAM are indicated below the sequence. (**b**) Western blot analysis of canonical autophagy. The indicated MEFs were starved for the indicated times, and expression of the indicated proteins was analyzed by western blotting. Actin was included as a loading control. (**c**) The indicated MEFs expressing GFP-LC3 were starved for the indicated times and GFP-LC3 puncta formation was analyzed. Representative images are shown. Bars = 10 μm. Deletion of *wipi2* showed a substantial reduction in canonical autophagy. Source data are provided as a Source Data file.

Suppl. Fig. 16 Yamaguchi et al.

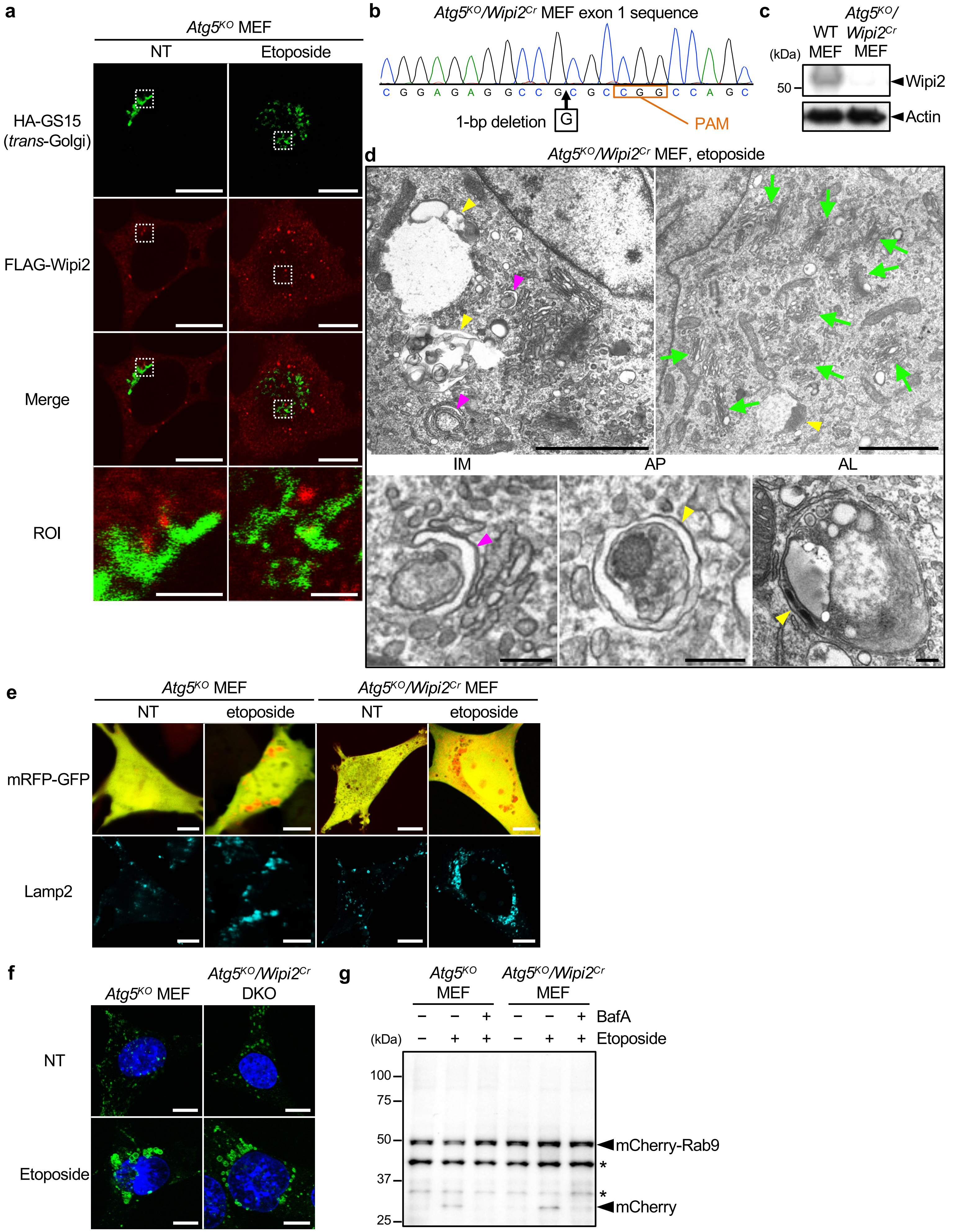

Supplementary Figure 16. *Wipi2* deficiency has no effects on alternative autophagy

(**a**) Wipi2 does not localized to the *trans*-Golgi. $Atg5^{KO}$ MEFs expressing Flag-Wipi2 and HA-GS15 were left untreated (NT) or were treated with etoposide (10 µM) at 12 hr. Then, cells were immunostained and observed by confocal microscopy. Red and green signals indicate Flag-Wipi2 and HA-GS15 (*trans*-Golgi), respectively. Bars = 10 µm. ROIs are indicated by the dashed squares and magnified images are shown in the bottom panels. Bars = 2 µm. (**b**, **c**) $Atg5^{KO}/Wipi2^{Cr}$ MEFs were generated from $Atg5^{KO}$ MEFs using the CRISPR/Cas9 system. A 1-bp deletion in *Wipi2* (first exon) was confirmed by genomic sequencing. The deleted nucleotide sequence and PAM are indicated below the sequence. In (**c**), the deletion of Wipi2 was confirmed by western blot analysis. Actin was used as a loading control. (**d**) $Atg5^{KO}/Wipi2^{Cr}$ MEFs were treated with etoposide (10 µM for 12 hr), and analyzed using EM. Bars = 2 µm. Isolation membranes (magenta arrowheads), autophagic vacuoles (yellow arrowheads), and normal ministacked Golgi (green arrows) were observed. Representative autophagic structures are shown in the bottom panels. Bars =0.2 µm. (**e**) The indicated MEFs expressing the mRFP-GFP protein were left untreated (NT) or were treated with etoposide (10 µM; 18 hr). Then, cells were immunostained with an anti-Lamp2 antibody (blue). Bars =10 µm. Red signals surrounded by Lamp2 signals indicate autolysosomes, and they were observed at similar levels in $Atg5^{KO}$ MEFs and $Atg5^{KO}/Wipi2^{Cr}$ MEFs. (**f**) The indicated MEFs were left untreated (NT) or treated with etoposide (10 µM; 18 hr), and were stained with an anti-Lamp2 antibody. Bars = 10 µm. Ring-like Lamp2 puncta were equivalently observed in $Atg5^{KO}$ MEFs and $Atg5^{KO}/Wipi2^{Cr}$ MEFs. (**g**) The mCherry-Rab9 cleavage assay was performed by the same method as in Fig. 2g, using the indicated MEFs. Asterisks indicate nonspecific bands. Both cells showed similar levels of autophagic cleavage. Source data are provided as a Source Data file.

Suppl. Fig. 17 Yamaguchi et al.

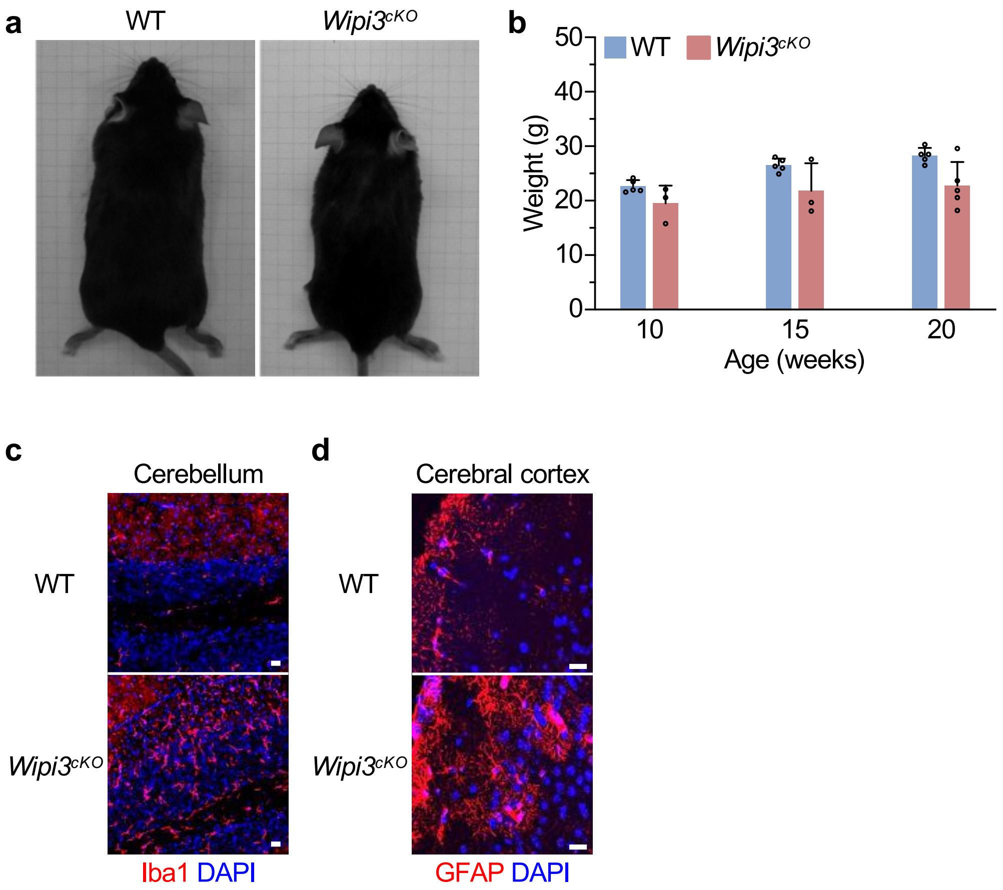

Supplementary Figure 17. Defects in neuron-specific *Wipi3^{cKO}* mice

(**a**, **b**) *Wipi3^{cKO}* mice have lower body weights than WT mice. (**a**) Photograph of a representative *Wipi3^{cKO}* mouse and a control mouse at 20 weeks of age. (**b**) Graph of body weights measured at the indicated ages. Data are shown as the mean ± SD (WT: n = 3; *Wipi3^{cKO}*, 10 weeks and 15 weeks: n = 3, *Wipi3^{cKO}*, 20 weeks: n = 5). Comparisons were performed using one-way ANOVA followed by the Tukey post-hoc test. (**c**, **d**) Immunohistochemical analyses of *Wipi3^{cKO}* mice. Cryosections of the cerebellum (**c**) and cerebral cortex (**d**) from the indicated mice were immunostained with the indicated antibodies. An increase in staining of the microglial marker Iba1 (**c**) and the glial marker GFAP (**d**) was observed. Bars = 20 µm. Source data are provided as a Source Data file.

Suppl. Fig. 18 Yamaguchi et al.

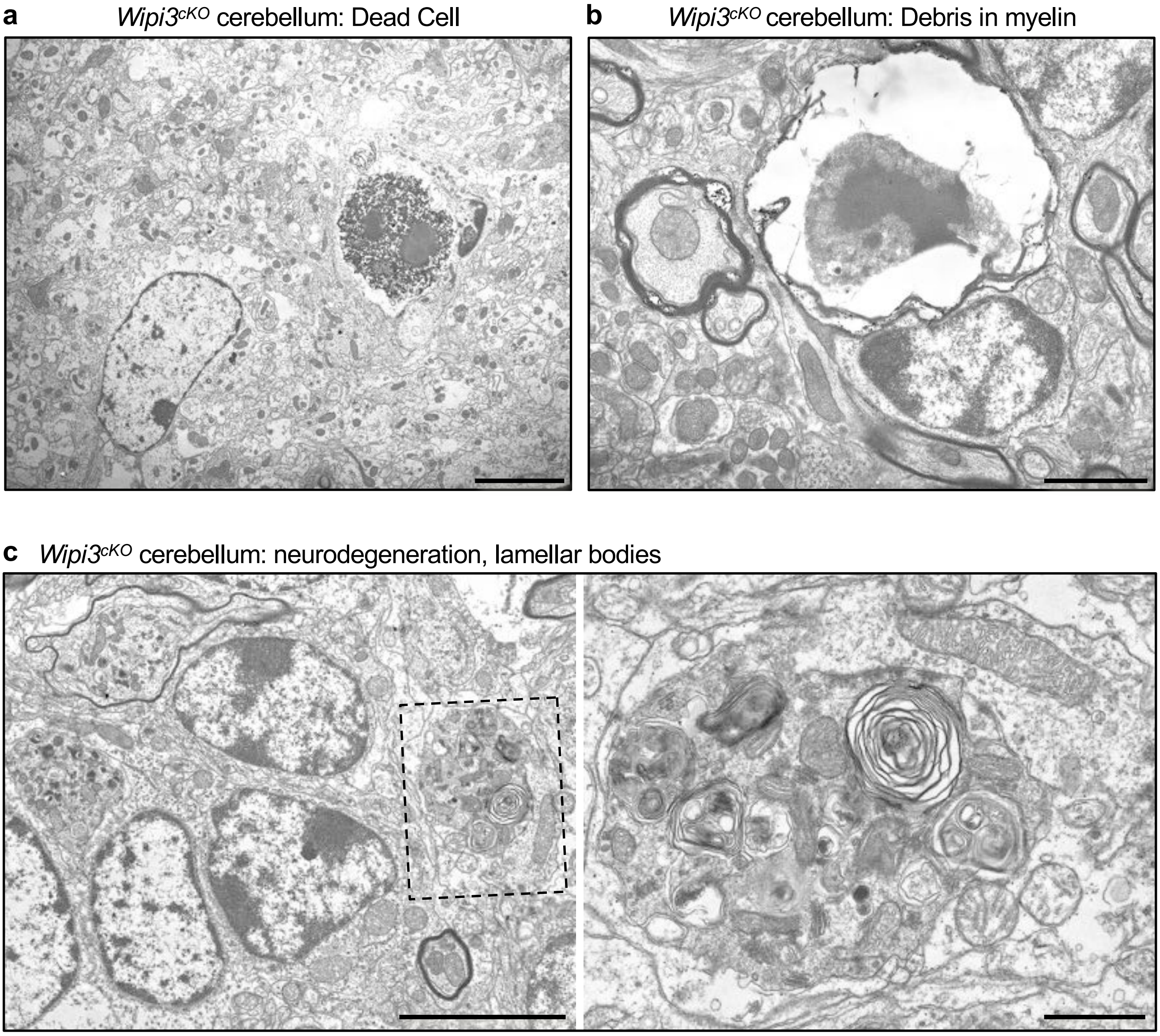

Supplementary Figure 18. Electron micrographs of cerebella from *Wipi3^{cKO}* mice

Representative images of dead cells (**a**), a collapsed nerve fiber in myelin (**b**), and lamellar bodies (**c**) are shown. Bars = 5 µm in (**a**, **c**; left panel), and 2 µm in (**b**). In (**c**), magnified images of the dashed square is shown in the right panel; bar = 1 µm.

Suppl. Fig. 19 Yamaguchi et al.

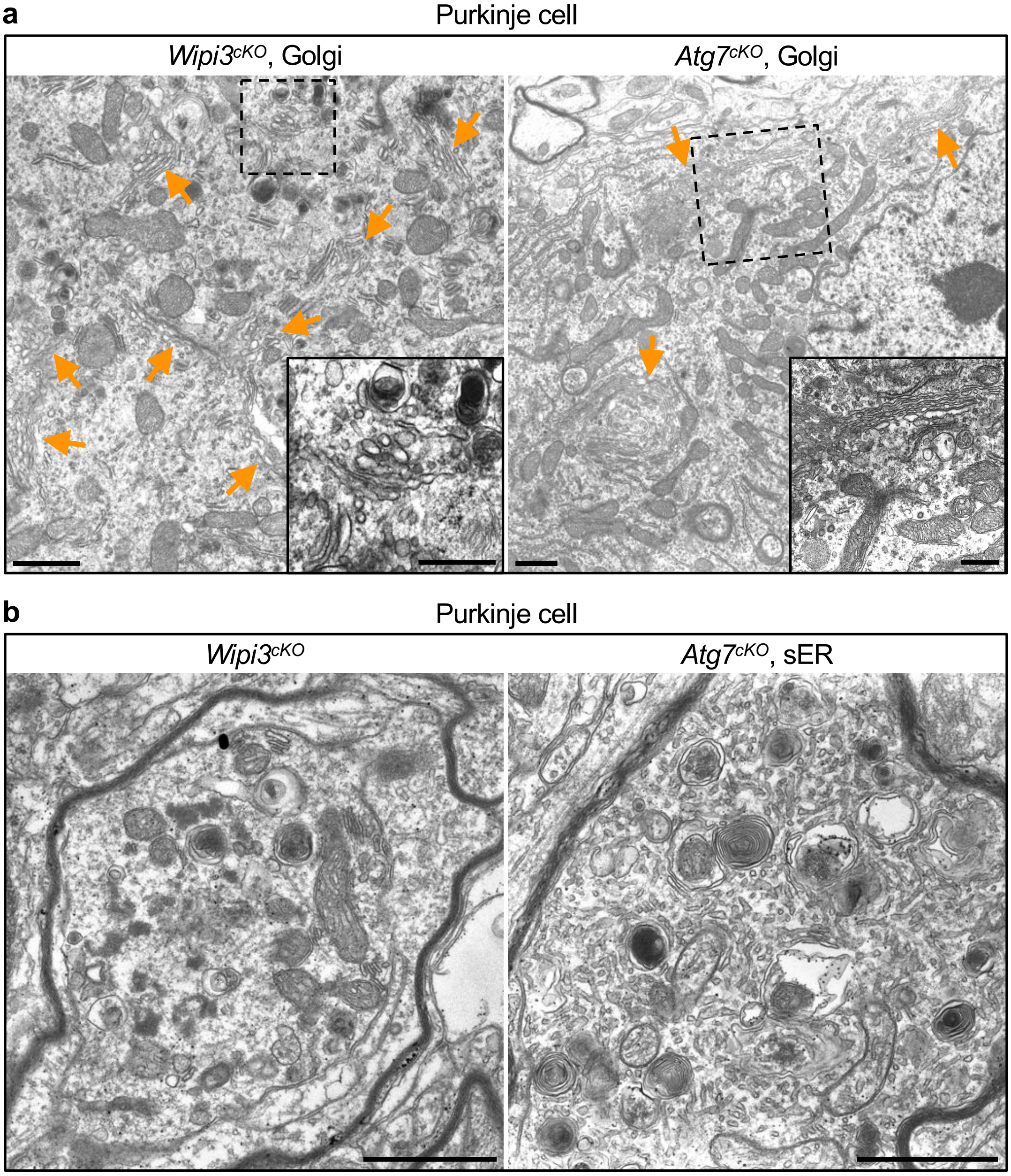

Supplementary Figure 19. Morphology of the Golgi and ER of *Wipi3^{cKO}* mice and *Atg7^{cKO}* mice
Electron micrographs of Purkinje cells from the indicated mice. (**a**) Abnormal Golgi structures were observed in the Purkinje cells from *Wipi3^{cKO}* mice, but not *Atg7^{cKO}* mice. Bars = 1 μm. Magnified images of the dashed squares are shown in the inset. Bars = 0.5 μm. Arrows indicate the Golgi membranes. (**b**) Enrichment of the smooth ER was observed in the Purkinje cells from *Atg7^{cKO}* mice, but not *Wipi3^{cKO}* mice. Bars = 1 μm.

Suppl. Fig. 20 Yamaguchi et al.

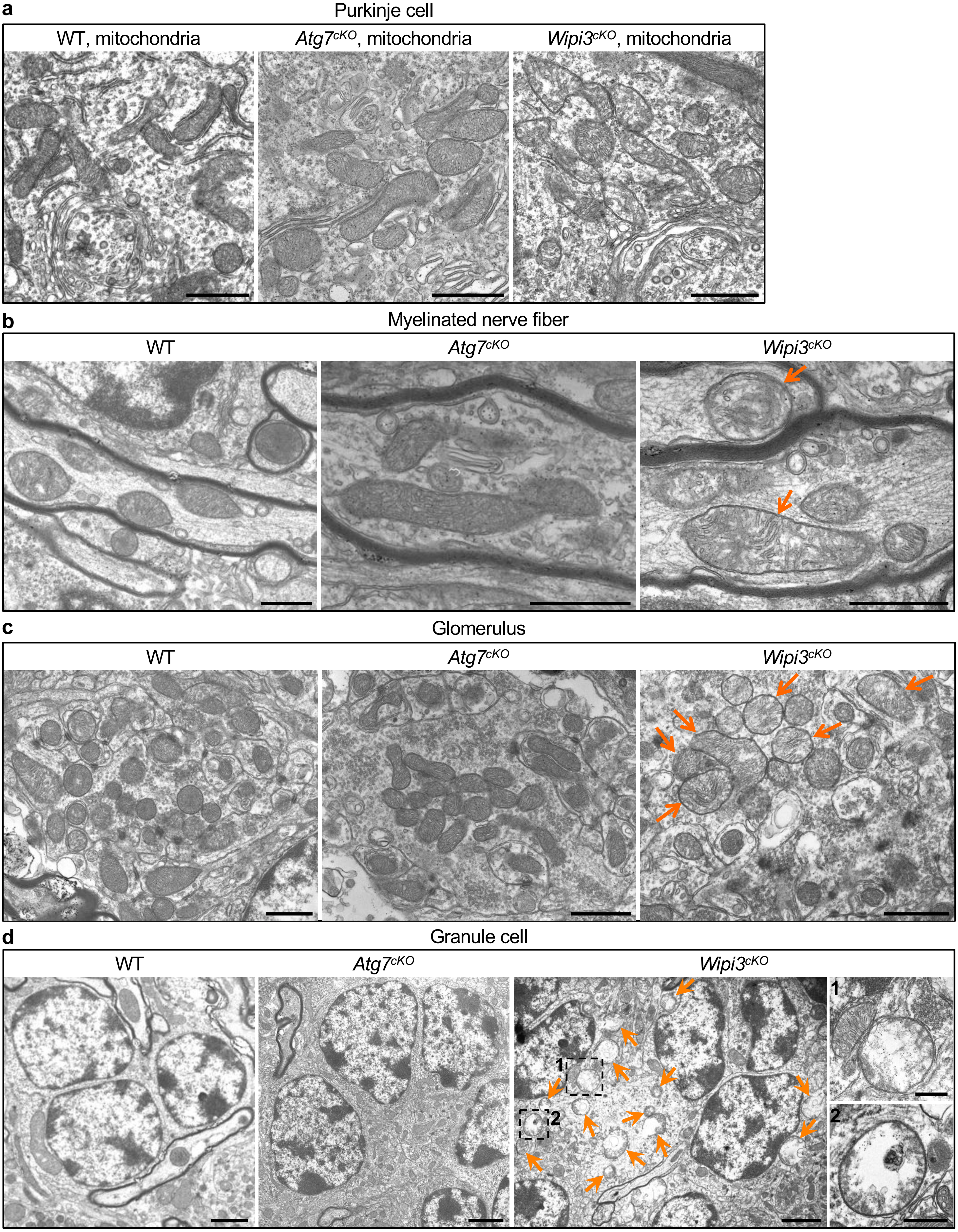

Supplementary Figure 20. Morphology of mitochondria from *Wipi3*^{cKO} mice and *Atg7*^{cKO} mice

Swollen mitochondria were observed in Purkinje cells (**a**), myelinated nerve fibers (**b**), glomeruli (**c**), and granule cells (**d**) from *Wipi3*^{cKO} mice, but not from WT mice and *Atg7*^{cKO} mice. Arrows indicate swollen mitochondria. Bars = 1 μm in (**a** to **c**) and 2 μm in (**d**). In (**d**), magnified images of the square are shown in the right panels; bar = 0.5 μm.

Suppl. Fig. 21 Yamaguchi et al.

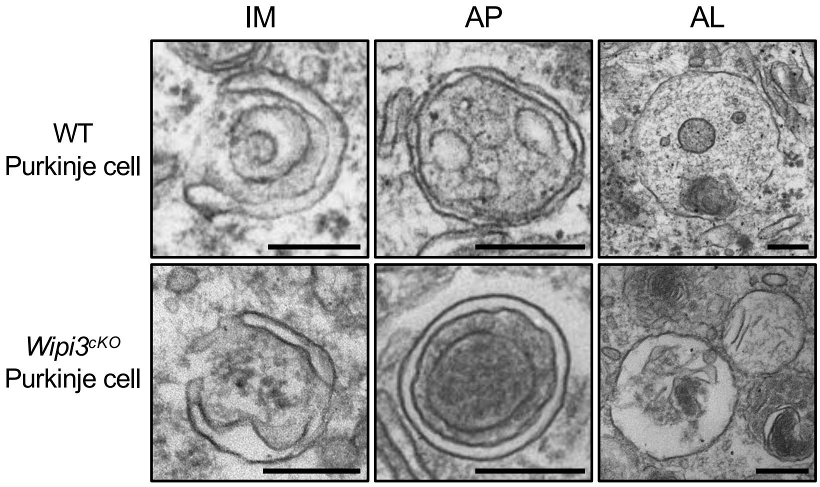

Supplementary Figure 21. Presence of a small number of autophagic structures in *Wipi3^{cKO}* mice

A representative isolation membrane (IM), autophagosome (AP), and autolysosome (AL) were observed in the Purkinje cells of WT mouse and *Wipi3^{cKO}* mouse. Bars = 0.2 μm.

Suppl. Fig. 22 Yamaguchi et al.

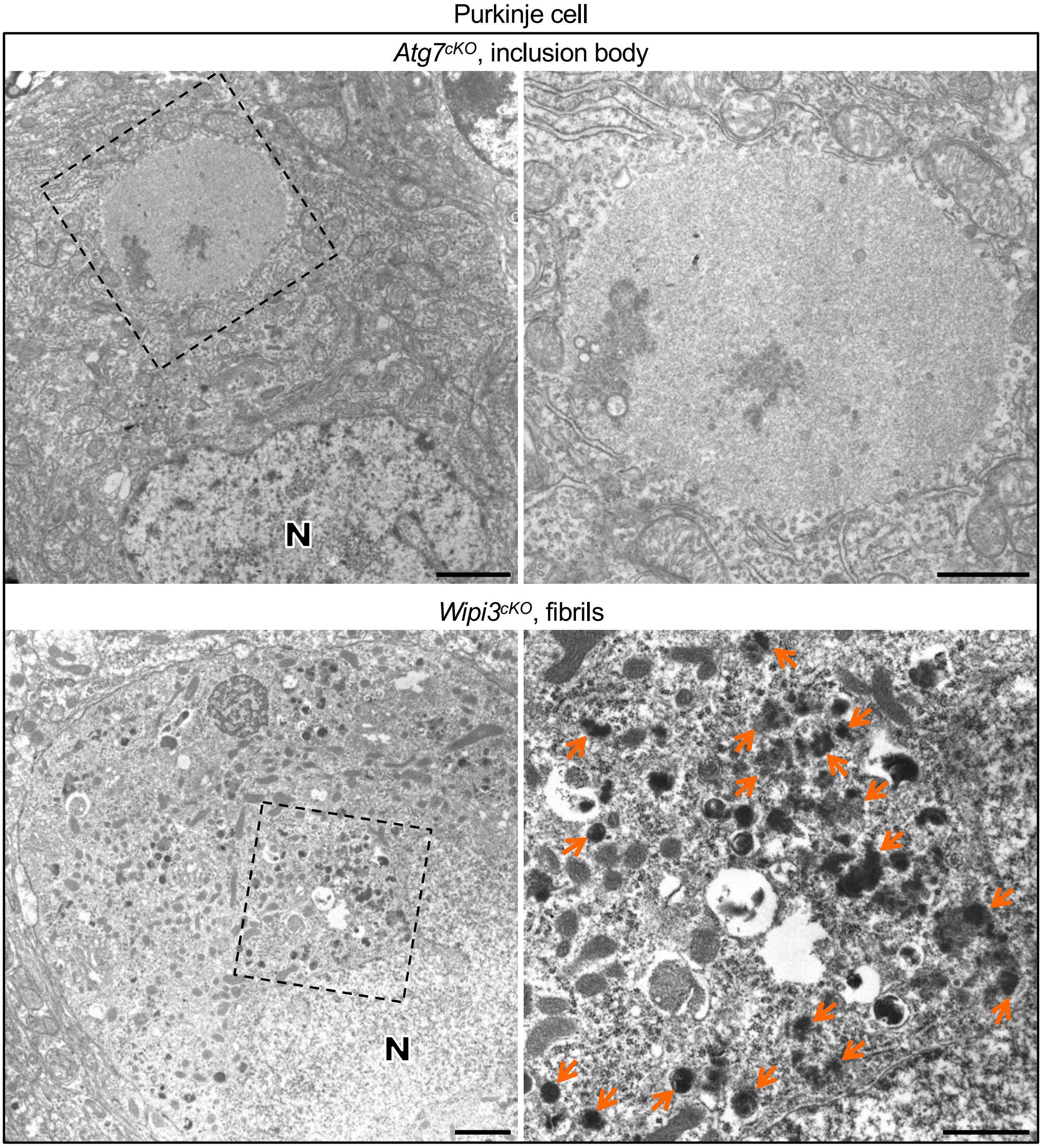

Supplementary Figure 22. Electron micrographs of Purkinje cells from *Wipi3^{cKO}* mice and *Atg7^{cKO}* mice
Formation of an inclusion body and dense fibrils in Purkinje cells from *Atg7^{cKO}* mice and *Wipi3^{cKO}* mice, respectively, which are the most prominent abnormal structures observed in these mice. Magnified images of the dashed squares are shown in the right panels. Arrows and "N" indicate dense fibrils and nucleus, respectively. Bars = 2 µm (left panels) and 1 µm (right panels).

Suppl. Fig. 23 Yamaguchi et al.

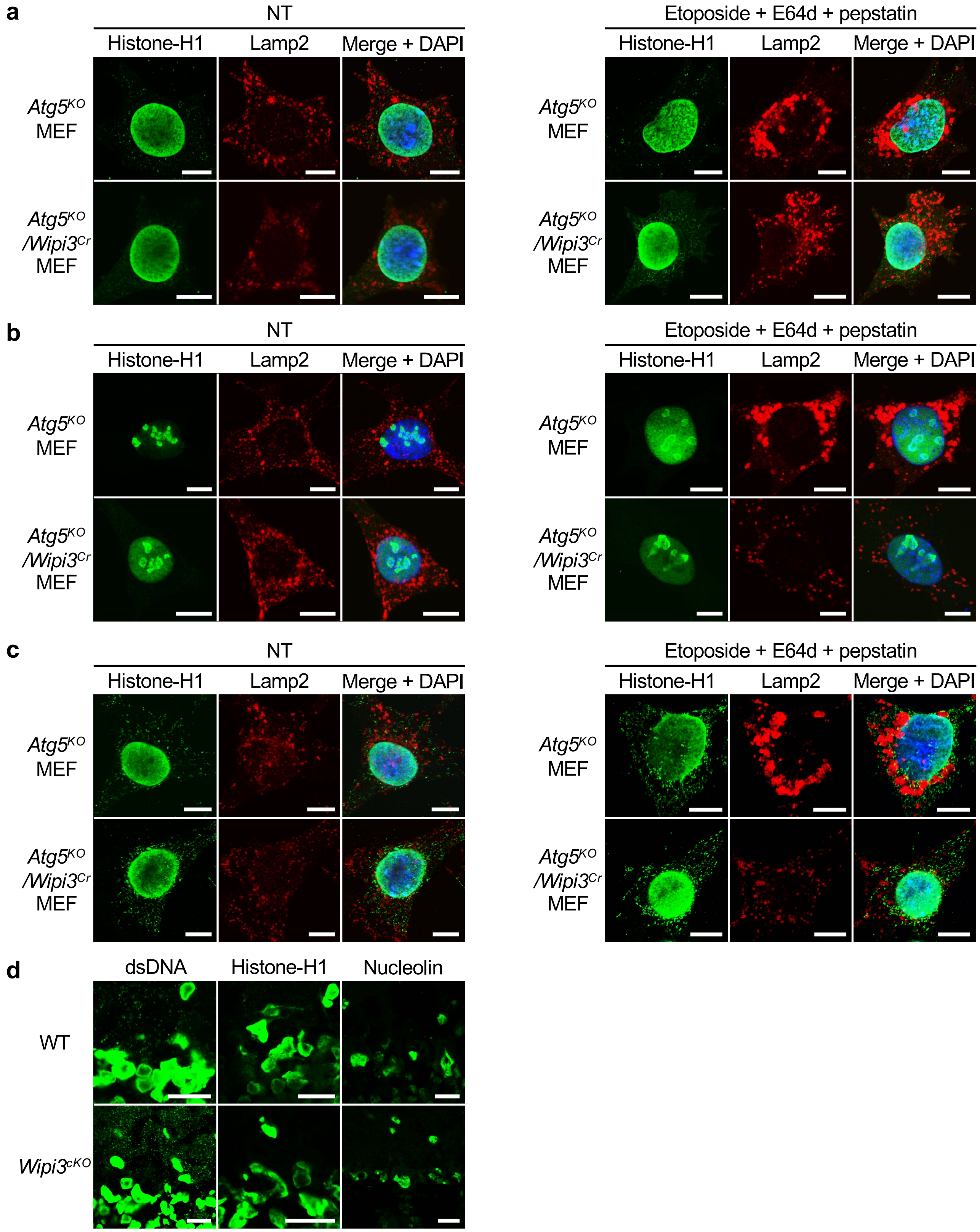

Supplementary Figure 23. Wipi3 deficiency has little effect on nucleophagy

(**a**, **b**) The indicated MEFs were left untreated (NT) or treated with etoposide (10 µM). We also added E64d and pepstatin to visualize cargos in autolysosomes. After 18 hr, cells were stained with anti-Histon-H1 (linker histone) or anti-nucleolin (ubiquitously distributed protein in the nucleolus) antibodies together with anti-Lamp2 antibody. Large Lamp2 puncta (autolysosomes) were observed in *Atg5^{KO}* MEFs, but not *Atg5^{KO}/Wipi3^{Cr}* MEFs. We did not observe engulfed Histon-H1 and nucleolin into autolysosomes. Nuclear morphology was also appeared normal. (**c**) The indicated MEFs were left untreated (NT) or treated with etoposide (10 µM). After 18 hr, cells were stained with anti-dsDNA and anti-Lamp2 antibodies. We did not observe engulfed dsDNA into autolysosomes. (**d**) The indicated cerebellum were stained with the indicated antibodies. These antibodies stained nuclei and they looked normal. We did not observe any differences between these two mice. Bars = 10 µm.

Suppl. Fig. 24 Yamaguchi et al.

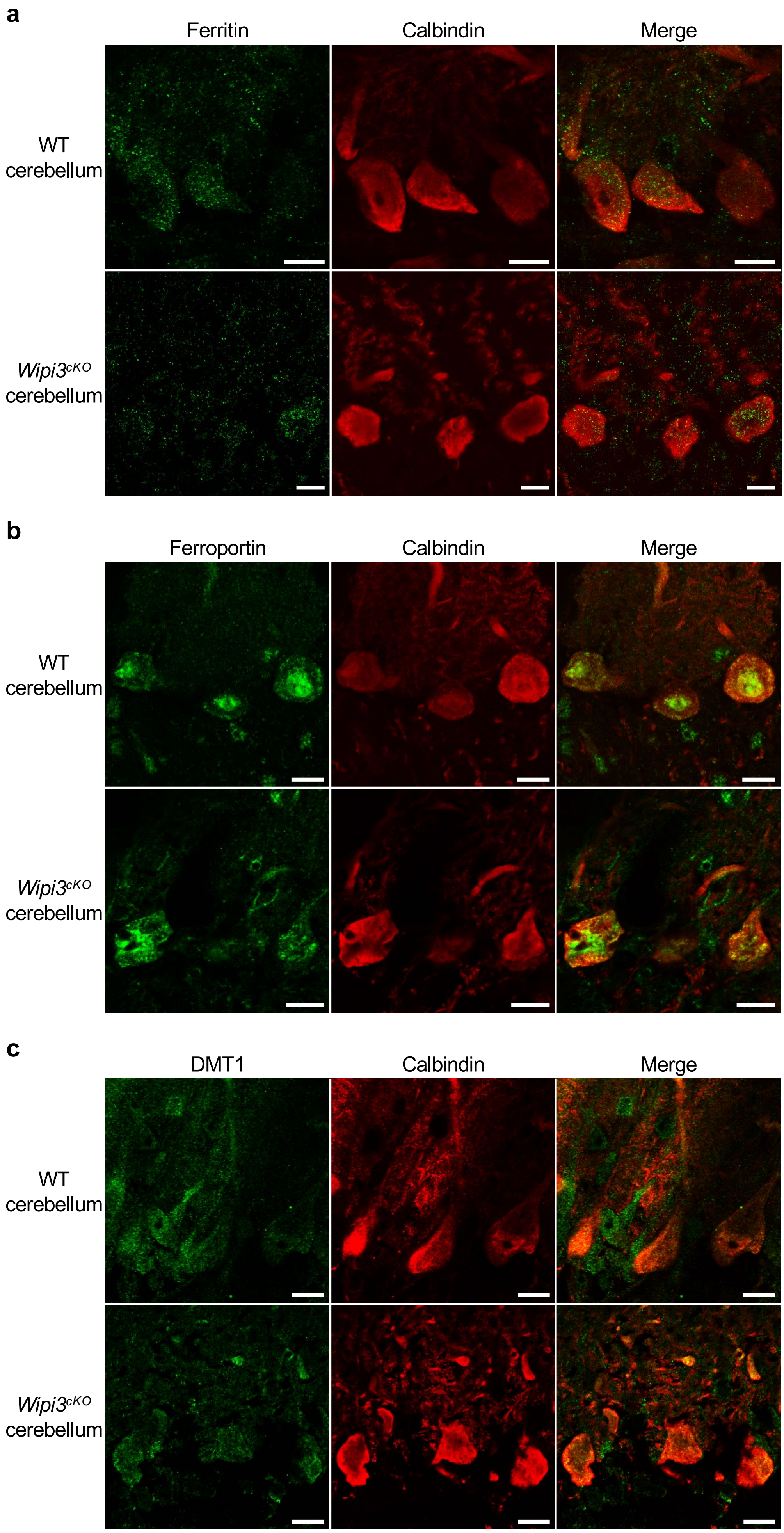

Supplementary Figure 24. Equivalent expression levels of ferritin, ferroportin, and DMT1 in *Wipi3*^{cKO} mice and WT mice
Cryosections of the cerebellum from *Wipi3*^{cKO} mice and WT mice were co-immunostained with anti-ferritin (**a**), anti-ferroportin (**b**), anti-DMT1 (**c**). Purkinje cells were counterstained by anti-calbindin antibodies (red). Bars = 10 μm.

Suppl. Fig. 25 Yamaguchi et al.

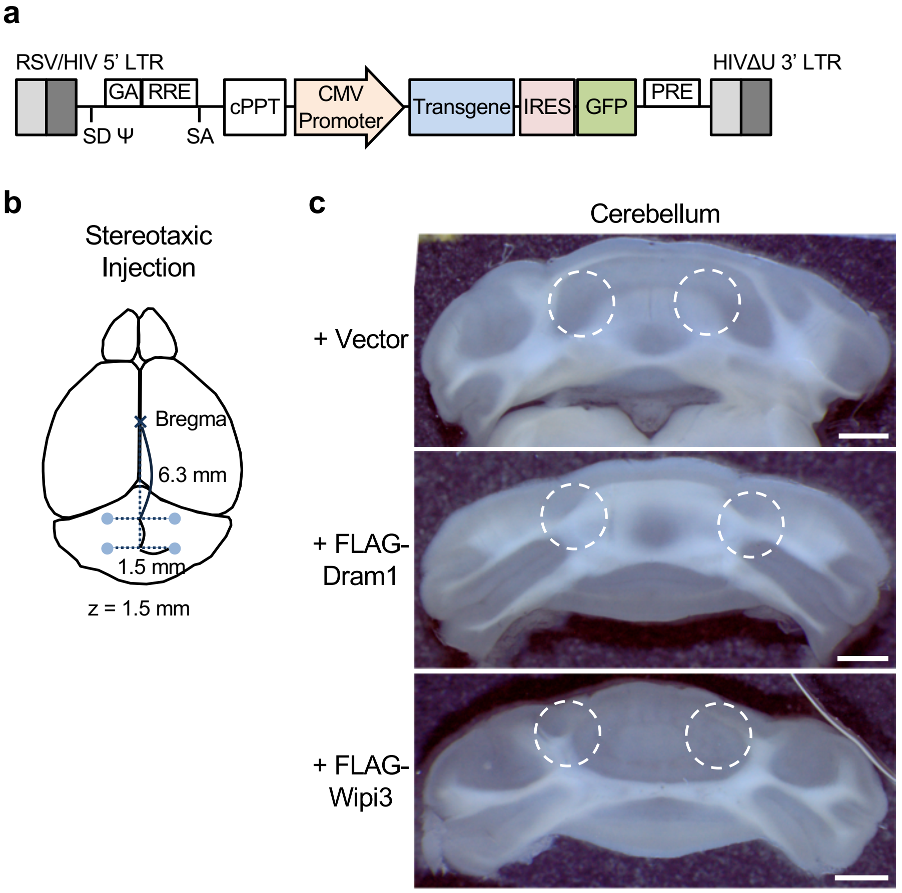

Supplementary Figure 25. Schematic view of the lentivirus injections

Lentivirus constructs containing a CMV promoter driving Flag-Dram1 or Flag-Wipi3 expression with IRES-GFP were injected into the mouse cerebellum. (**a**) The lentivirus construct used. (**b**) A schematic view of the lentivirus injections. The blue spots indicate the stereotaxic injection sites. (**c**) Representative images of the cerebellum from each mouse are shown. Specimens for EM were obtained from the areas indicated by the dashed circles. Bars = 1 mm.

Suppl. Fig. 26 Yamaguchi et al.

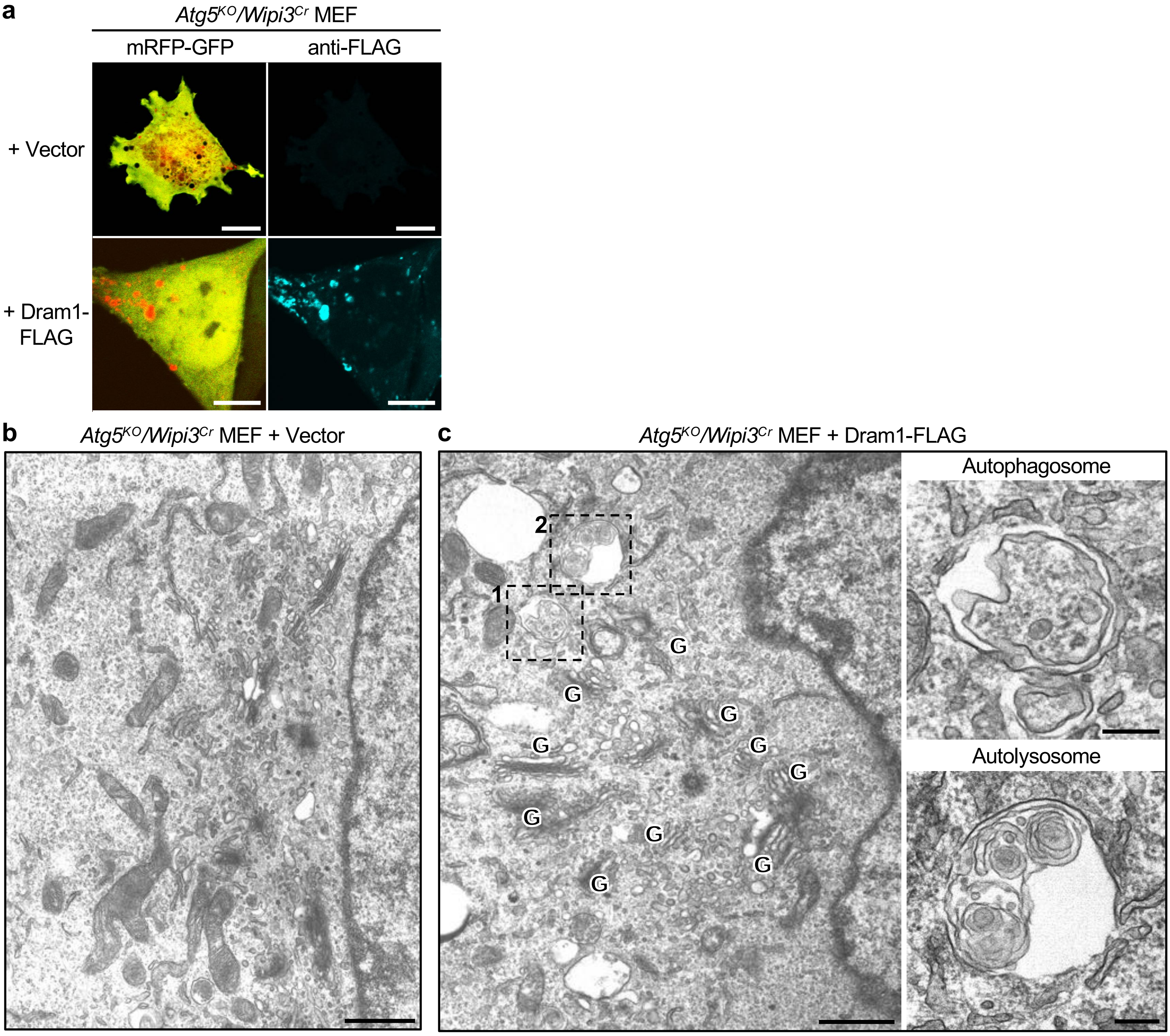

Supplementary Figure 26. Induction of alternative autophagy in *Atg5^{KO}/Wipi3^{Cr}* MEFs by Dram1
Atg5^{KO}/Wipi3^{Cr} MEFs expressing a mRFP-GFP tandem protein were transfected with a vector control or the Dram1-flag plasmid. After 18 hr, the cells were immunostained with an anti-Flag antibody, and observed using confocal microscopy. (**a**) Representative images are shown. Red puncta indicate acidic compartments and were merged with Flag-Dram1 (blue). Bars = 10 μm. Expression of Dram1 induced alternative autophagy. (**b**, **c**) Electron micrographs of vector-transfected and Dram1-transfected *Atg5^{KO}/Wipi3^{Cr}* MEFs. The indicated plasmids were transfected into *Atg5^{KO}/Wipi3^{Cr}* MEFs, and analyzed after 24 hr using EM. (**b**) In empty vector-transfected *Atg5^{KO}/Wipi3^{Cr}* MEFs, no obvious abnormalities were observed. Bars = 1 μm. (**c**) In Dram1-transfected *Atg5^{KO}/Wipi3^{Cr}* MEFs, autophagic structures were generated. Autophagic structures are indicated by the dashed squares and magnified images are shown in the right panels. "G" indicates the Golgi apparatus. Bars = 1 μm (left panel) and 0.2 μm (right panels).

Suppl. Fig. 27 Yamaguchi et al.

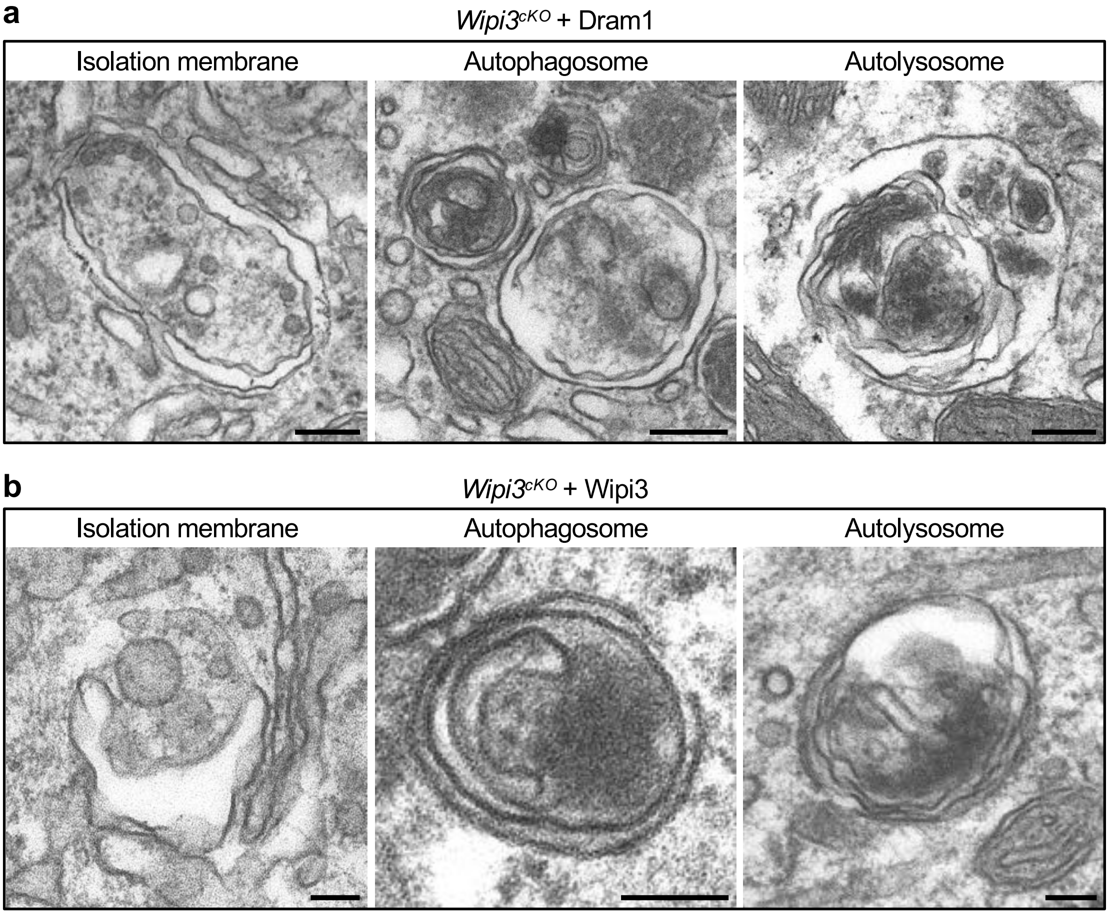

Supplementary Figure 27. Electron micrographs of Purkinje cells from Wipi3-transfected and Dram1-transfected *Wipi3^{cKO}* mice

Representative autophagic structures in Purkinje cells of Dram1-transfected *Wipi3^{cKO}* mouse (**a**) and Wipi3-transfected *Wipi3^{cKO}* mouse (**b**). Autophagic structures degrading dense fibrils were observed. Bars = 0.2 µm in (**a**), 0.1 µm in (**b**).

Suppl. Fig. 28 Yamaguchi et al.

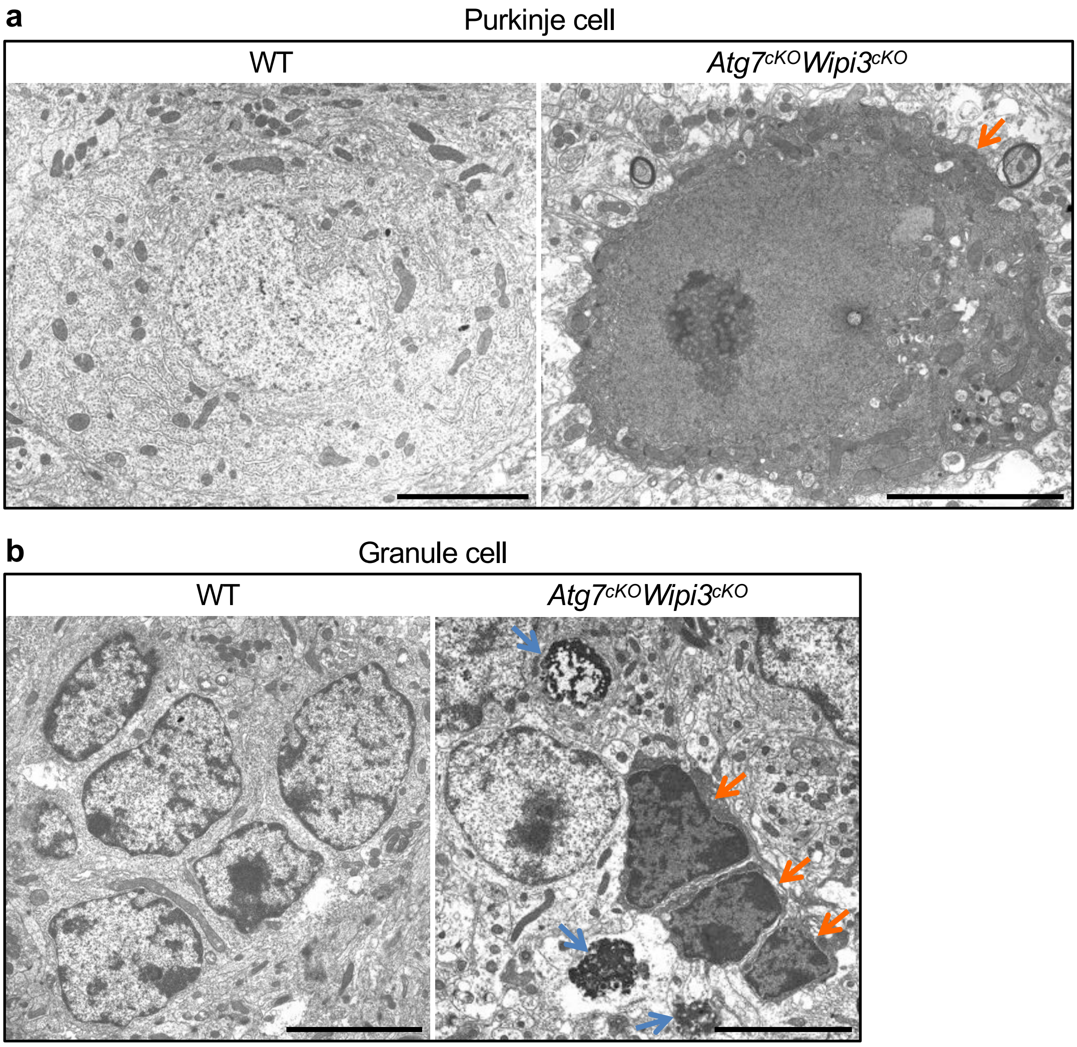

Supplementary Figure 28. Electron micrographs of cerebella from *Atg7^{cKO}/Wipi3^{cKO}* mice

Electron micrographs of Purkinje cells (**a**) and granular cells (**b**) from the indicated mice. Many dead cells (orange arrows) and debris (blue arrows) were observed in cerebella from *Atg7^{cKO}/Wipi3^{cKO}* mice. Bars = 5 μm.

Suppl. Fig. 29 Yamaguchi et al.

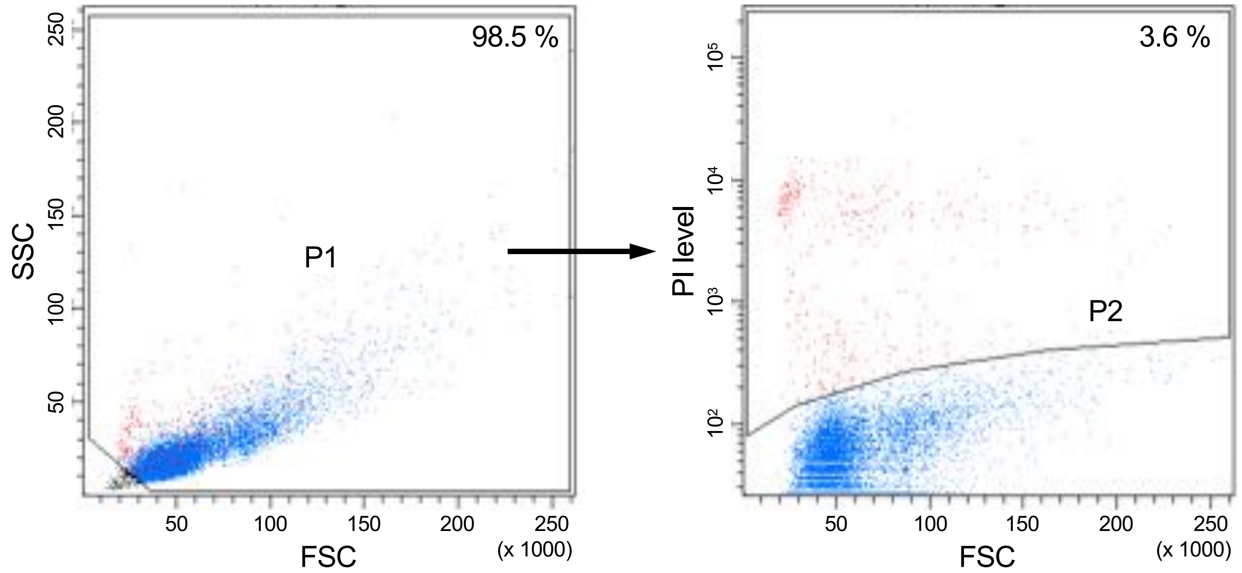

Supplementary Figure 29. Flow Cytometry gating strategies

For cell viability assay, cellular debris was removed using FSC/SSC (left panel). Then, PI-positive dead cells were detected using FSC/PI (right panel).

Supple. Table 1 Yeast Strain List

Strain	Genotype	Reference or source
SEY6210	*MATalpha leu2-3,112 ura3-52 his3-Δ200 trp1-Δ901 lys2-801 suc2-Δ9*	Robinson et al. (1998)
HY526	SEY6210; *atg5Δ::URA3 pep4Δ::TRP1*	This study
HY631	SEY6210; *atg5Δ::HIS3 pho8::pTDH3-GFP-pho8Δ60-URA3*	This study
HY1161	SEY6210; *atg5Δ::URA3 hsv2Δ::HIS3 pep4Δ::TRP1*	This study
HY1343	SEY6210; *atg5Δ::HIS3 hsv2Δ::LEU2 pho8::pTDH3-GFP-pho8Δ60-TRP1*	This study

Suppl. Table 2 Plasmid List

Plasmid	Description	Source
pHYA877	pMSCVhygro-mCherry-mRab9a	This Study
pHYA2451	pCAGGS-VSVG^{ts045}-GFP	This Study
pHYA2473	pcDNA3.1Hygro(+)-GFP-rLC3	This Study
pHYA2529	pcDNA3.1Hygro(+)-mRFP-GFP	This Study
pHYA2592	pcDNA3.1Hygro(+)-mCherry-2xFYVE	This Study
pHYA2758	pcDNA3.1Hygro(+)-19xHA-rLC3	This Study
pHYA2882	pcDNA3.1Hygro(+)-mRFP-GFP-mRab9a	This Study
pHYA2896	pMSCVhygro-3xFLAG-Linker24-mWipi3	This Study
pHYA2899	pcDNA3.1Hygro(+)-3xFLAG-Linker24-mWipi3	This Study
pHYA2932	pMSCVhygro-3xFLAG-Linker24-mWipi3 R225A	This Study
pHYA2933	pMSCVhygro-3xFLAG-Linker24-mWipi3 R226A	This Study
pHYA2937	pcDNA3.1Hygro(+)-3xFLAG-Linker24-mWipi3 R225A	This Study
pHYA2938	pcDNA3.1Hygro(+)-3xFLAG-Linker24-mWipi3 R226A	This Study
pHYA2963	pcDNA3.1Hygro(+)-3xHA-mGS15	This Study
pHYA3006	pcDNA3.1Hygro(+)-3xFLAG-Linker24-mWipi2	This Study
pHYA3008	p3xFLAG-CMV-14-hDram1-3xFLAG	This Study
pHYA3063	pcDNA3.1Hygro(+)-mStrawberry-2xML1N	This Study
pHYA3071	pRRL-cPPT-CMV-X2-PRE-SIN-IRES-GFP	This Study
pHYA3076	pRRL-cPPT-CMV-X2-PRE-SIN-IRES-GFP-hDram1-3xFLAG	This Study
pHYA3077	pRRL-cPPT-CMV-X2-PRE-SIN-IRES-GFP-3xFLAG-Linker24-mWipi3	This Study
pHYA3113	pGEX4T-2-3xFLAG-Linker24-mWipi3	This Study
pHYA3114	pGEX4T-2-3xFLAG-Linker24-mWipi3 R225A	This Study
pHYA3115	pGEX4T-2-3xFLAG-Linker24-mWipi3 R226A	This Study
pHYA3145	pGEX4T-2-3xHA-2xFYVE	This Study
pHYA3186	pcDNA3.1Hygro(+)-GFP-Ceruloplasmin	This Study

Suppl. Table 3 Primer List

Names	Sequences
PHO8-d60-EcoRI-F	ttcGAATTCatgggaacttctgcatcacacaagaag
PHO8-d60-R	gacgtcgactgcgctgtgacc
ATG5-1429F	tgcaattcacactgacggag
ATG5-3530R	tagcaggagtgggagttga
p4-F-BamHI	tccggatccgcagcatagaacaatggattc
p4-R-PstI	cagctgcagcctcaattgtatttgctgagg
HSV2-4205-F	ctgactattcgctcgaaatggacg
HSV2-6483-R	caatgctatcaaagatgcgtacaag
Rab9-ORF-BamHI-F	tccGGATCCatggcaggaaaatcgtctc
Rab9-ORF-XhoI-R	gagCTCGAGtcaacagcaagatgagtttgg
VSVG-ORF-EcoRI-F	ttcGAATTCatgaagtgccttttgtacttagc
pEGFP-VSVG-EcoRI-R	ttcGAATTCaattctgtttccaagtcggttcatc
BamHI-mRFP-F	tccGGATCCatggcctcctccgaggacgtcatc
T3-R	attaaccctcactaaagggaa
FYVE-BamHI-F	tccGGATCCgaaagtgatgccatgttcgc
FYVE-linker-BamHI-R	ccGGATCCTTGACCTTGtgccttcttgttcagctgctc
FYVE-BamHIBclI-F	tccGGATCCTGATCAgaaagtgatgccatgttcgc
FYVE-ter-BamHI-R	tccGGATCCTCAtgccttcttgttcagctgctc
mWIPI3-ORF-BglII-F2	gatctAGATCTaacctcctgccgtgtaaccctcac
mWIPI3-ORF-XhoI-R2	tcgagCTCGAGtcacagcttgtcatcggtcatctc
mWIPI3-ORF-R225A-F	ttaatccaggagctaGCaagaggatctcaagc
mWIPI3-ORF-R225A-R	gcttgagatcctcttGCtagctcctggattaa
mWIPI3-ORF-R226A-F	ccaggagctacgaGCaggatctcaagcagc
mWIPI3-ORF-R226A-R	gctgcttgagatcctGCtcgtagctcctgg
mGS15-BglII-F	tctAGATCTgcagattggactcgagctcagag
mGS15-EcoRI-R	gattcGAATTCtcacgtccttgtcctcgacaagag
mWIPI2-ORF-BamHI-F2	tccGGATCCaacctggcgagccagagcggag
mWIPI2-BamHI-1-F	tccggatccatgaacctggcgagccagagcggag
mWIPI2-SalI-1338-R	ggagtcgactcagtcagtccggagaatcatgg
CMV-F	cgcaaatgggcggtaggcgtg
pHYA3008-1756-EcoRI-R	gattcGAATTCcacccgggatcactacttg
mCp-ORF(109)-BamHI-F	tccGGATCCaagtttttgctgcttagcaca
mCp-ORF(109)-XhoI-R	ctctCTCGAGtcagccagacttagtctcttg
mWIPI3-33819-F	caactaagggcatgatgcctgc
mWIPI3-34980-R	ggatagccagtggtgttacacag
mATG5-21282-F	catctgttaccctagctctac
mATG5-21775-R	ctgcttcagcctcttgaatgc
mWIPI2-1661-BamHI-F	tccGGATCCggtaactcgccgttctcag
mWIPI2-2499-R	agttacttgccagaaaggtcg
Atg5 Genotyping 1	gaatatgaaggcacacccctgaaatg
Atg5 Genotyping 2	acaacgtcgagcacagctgcgcaagg
Atg5 Genotyping 3	gtactgcataatggtttaactcttgc
ATG7F_v6_Minus45bp_Fw	ggaactgtcccactatgagccatgtt
ATG7int_Rv	gaacccagctagaggcactg
ATG7FICCasVer6_Rv	cagagaccatcagcagctccaca
loxR	tgaactgatggcgagctcagacc
WIPI3-5'arm	gtagaagcaggtctgtcac
WIPI3-3'arm	aagccccactcctaatgc
