# Supplementary material for: Wipi3 is essential for alternative autophagy and its loss causes neurodegeneration
Source: Nat Commun. 2020 Oct 20;11:5311. doi: 10.1038/s41467-020-18892-w (PMC7576787; doi:10.1038/s41467-020-18892-w)
Supplement: Supplementary file 3 — Reporting Summary [file 41467_2020_18892_MOESM3_ESM.pdf]

nature research

Corresponding author(s): Aurelio A. Teleman

Last updated by author(s): Sep 16, 2020

Reporting Summary

Nature Research wishes to improve the reproducibility of the work that we publish. This form provides structure for consistency and transparency in reporting. For further information on Nature Research policies, see our Editorial Policies and the Editorial Policy Checklist.

Statistics

For all statistical analyses, confirm that the following items are present in the figure legend, table legend, main text, or Methods section.

n/a	Confirmed	
☐	☒	The exact sample size (*n*) for each experimental group/condition, given as a discrete number and unit of measurement
☐	☒	A statement on whether measurements were taken from distinct samples or whether the same sample was measured repeatedly
☐	☐	The statistical test(s) used AND whether they are one- or two-sided<br>*Only common tests should be described solely by name; describe more complex techniques in the Methods section.*
☒	☐	A description of all covariates tested
☐	☒	A description of any assumptions or corrections, such as tests of normality and adjustment for multiple comparisons
☐	☒	A full description of the statistical parameters including central tendency (e.g. means) or other basic estimates (e.g. regression coefficient) AND variation (e.g. standard deviation) or associated estimates of uncertainty (e.g. confidence intervals)
☐	☒	For null hypothesis testing, the test statistic (e.g. *F*, *t*, *r*) with confidence intervals, effect sizes, degrees of freedom and *P* value noted<br>*Give P values as exact values whenever suitable.*
☒	☐	For Bayesian analysis, information on the choice of priors and Markov chain Monte Carlo settings
☒	☐	For hierarchical and complex designs, identification of the appropriate level for tests and full reporting of outcomes
☒	☐	Estimates of effect sizes (e.g. Cohen's *d*, Pearson's *r*), indicating how they were calculated

Our web collection on statistics for biologists contains articles on many of the points above.

Software and code

Policy information about availability of computer code

Data collection	Software that runs the BioRad Chemidoc and the Leica confocal microscope are commercially available.
Data analysis	ApE - A plasmid Editor for analysis of sequencing results and cloning - is freely available online; Bruker Daltonics FlexAnalysis 3.2 and Mascot Server for analysis of mass spectrometry data are commercially available; Cas-OFFinder and Optimized CRISPR Design were used to design sgRNAs are freely available online (http://www.rgenome.net/cas-offinder/); Graph Pad Prism 8 for statistics is commercially available; Image Lab Software was used to quantify chemiluminiscence on Western blots - commercially available; Primer-BLAST was used to design primers - available online at https://www.ncbi.nlm.nih.gov/tools/primer-blast/; GIMP 2.10 (freely available online) and Adobe Photoshop 2020 (commercially available) for figure analysis and preparation.

For manuscripts utilizing custom algorithms or software that are central to the research but not yet described in published literature, software must be made available to editors and reviewers. We strongly encourage code deposition in a community repository (e.g. GitHub). See the Nature Research guidelines for submitting code & software for further information.

Data

Policy information about availability of data

All manuscripts must include a data availability statement. This statement should provide the following information, where applicable:

- Accession codes, unique identifiers, or web links for publicly available datasets
- A list of figures that have associated raw data
- A description of any restrictions on data availability

Mass spectrometry data has been deposited at PRIDE (https://www.ebi.ac.uk/pride/).

Field-specific reporting

Please select the one below that is the best fit for your research. If you are not sure, read the appropriate sections before making your selection.

☒ Life sciences ☐ Behavioural & social sciences ☐ Ecological, evolutionary & environmental sciences

For a reference copy of the document with all sections, see nature.com/documents/nr-reporting-summary-flat.pdf

Life sciences study design

All studies must disclose on these points even when the disclosure is negative.

Sample size	The selection of sample size for each type of experiment in this study was based on previous experiments or preliminary testing. No statistical method was used to determine sample size.
Data exclusions	No data were excluded from analyses.
Replication	All experiments were repeated at least 2 times (biological replicates) and at least 3 times (biological replicates) for calculation of statistical significance.
Randomization	No randomization method was used.
Blinding	Data collection and analysis were not blinded.

Reporting for specific materials, systems and methods

We require information from authors about some types of materials, experimental systems and methods used in many studies. Here, indicate whether each material, system or method listed is relevant to your study. If you are not sure if a list item applies to your research, read the appropriate section before selecting a response.

Materials & experimental systems

n/a	Involved in the study	
☐	☒	Antibodies
☐	☒	Eukaryotic cell lines
☒	☐	Palaeontology and archaeology
☒	☐	Animals and other organisms
☒	☐	Human research participants
☒	☐	Clinical data
☒	☐	Dual use research of concern

Methods

n/a	Involved in the study	
☒	☐	ChIP-seq
☒	☐	Flow cytometry
☒	☐	MRI-based neuroimaging

Antibodies

Antibodies used

The following primary antibodies were used in this study:
Rabbit anti-AKT; Cell Signaling Technology; Cat# 9272; Lot: 28.
Rabbit anti-caveolin-1 (clone D46G3); Cell Signaling Technology; Cat# 3267; Lot: 6.
Rabbit anti-CD71 (Transferrin receptor 1) (clone D7G9X); Cell Signaling Technology; Cat# 13113; Lot: 2.
Rabbit anti-c-Myc/N-Myc (clone D3N8F); Cell Signaling Technology; Cat# 13987; Lot: 5.
Rabbit anti-EGF receptor (clone D38B1); Cell Signaling Technology; Cat# 4267; Lot: 19.
Rabbit anti-flotillin-1 (clone D2V7J); Cell Signaling Technology; Cat# 18634; 1.
Rabbit anti-G protein alpha inhibitor 1 antibody (clone EPR94441(B)); Abcam; Cat# ab140125; Lot: GR102695-9.
Rabbit anti-G protein alpha inhibitor 2 antibody (clone EPR9468); Abcam; Cat# ab137050; Lot: YJ050309CS.
Rabbit anti-Gab1; Cell Signaling Technology; Cat# 3232; Lot: 7.
Rabbit anti-GAPDH (clone 14C10); Cell Signaling Technology; Cat# 2118; Lot: 14.
Guinea pig anti-GFP; produced in Teleman lab.
Mouse anti-GM130; BD Biosciences; Cat# 610823.
Rabbit anti-GNAI3 antibody (clone EPR11894); Abcam; Cat# ab173527; Lot: YK020621CS.
Rabbit anti-p44/42 MAPK (Erk1/2) (clone 3A7); Cell Signaling Technology; Cat# 9107; Lot: 10.
Rabbit anti-pan-cadherin (clone 28E12); Cell Signaling Technology; Cat# 4073; Lot: 1.
Mouse anti-PDI (clone 1D3); Abcam; Cat# ab190883; Lot: GR259862-2.
Rabbit anti-phospho-AKT (Ser473); Cell Signaling Technology; Cat# 9271; Lot: 14.
Rabbit anti-phospho-AKT (Thr308) (clone C31E5E); Cell Signaling Technology; Cat# 2965; Lot: 18.
Rabbit anti-phospho-p44/42 MAPK (Erk1/2) (Thr202/Tyr204) (clone D13.14.4E); Cell Signaling Technology; Cat# 4370; Lot: 17.
Mouse anti-V5 tag (clone P/N46-0705); ThermoFisher; Cat# R960-25; Lot: 1821026.

	Mouse anti-α-tubulin (clone DM1A); Sigma-Aldrich; Cat# T9026.<br><br>The following secondary antibodies were used for immunoblotting and confocal microscopy:<br>FITC-conjugated AffiniPure Donkey Anti-Mouse IgG (H+L); Jackson ImmunoResearch Labs Cat# 715-095-151.<br>HRP-conjugated AffiniPure Goat Anti-Rabbit IgG (H+L); Jackson ImmunoResearch Labs; Cat# 111-035-003.<br>TRITC-conjugated AffiniPure Donkey Anti-Rabbit IgG (H+L); Jackson ImmunoResearch Labs; Cat# 711-025-152.<br>HRP-conjugated AffiniPure Goat Anti-Guinea Pig IgG (H+L) ;Jackson ImmunoResearch Labs; Cat# 106-035-003.<br>HRP-conjugated AffiniPure Goat Anti-Mouse IgG (H+L); Jackson ImmunoResearch Labs; Cat# 115-035-003.
Validation	Validation statement of each primary antibody can be found on the manufacturer's website.<br>Isoform-specific validation of anti-GNAI1 (anti-G protein alpha inhibitor 1 antibody), anti-GNAI2 (anti-G protein alpha inhibitor 2 antibody) and anti-GNAI3 antibodies was performed via immunoblotting of lysates of cells with knocked down or overexpressed GNAI isoforms as shown in this manuscript.<br>Validation of anti-GFP antibody was performed via immunoblotting of lysates of cells overexpressing GFP.

Eukaryotic cell lines

Policy information about cell lines

Cell line source(s)	MCF7 cells were purchased from ATCC.
Authentication	The paternal and derived MCF7 cell lines used in this study were authenticated by Multiplexion (Heidelberg, Germany) as described in [PMID]: 22700458.
Mycoplasma contamination	All cell lines were tested negative for mycoplasma contamination.
Commonly misidentified lines<br>(See ICLAC register)	No commonly misidentified cell lines were used.
